# Tissue-mimetic culture enhances mesenchymal stem cell secretome capacity to improve regenerative activity of keratinocytes and fibroblasts in vitro

**DOI:** 10.1111/wrr.13076

**Published:** 2023-03-21

**Authors:** Jacob G. Hodge, Heather E. Decker, Jennifer L. Robinson, Adam J. Mellott

**Affiliations:** 1 Bioengineering Graduate Program, University of Kansas, Lawrence, Kansas, USA; 2 Department of Plastic Surgery, University of Kansas Medical Center, Kansas City, Kansas, USA; 3 Ronawk, LLC, Olathe, Kansas, USA; 4 Department of Chemical and Petroleum Engineering, University of Kansas, Lawrence, Kansas, USA

**Keywords:** biomaterials, hydrogels, regenerative medicine, secretome, stem cells, tissue engineering

## Abstract

Mesenchymal stem/stromal cells (MSCs) are a heterogenous population of multipotent and highly secretory cells currently being investigated in the field of wound healing for their ability to augment tissue responses. The adaptive response of MSC populations to the rigid substrate of current 2D culture systems has been considered to result in a deterioration of regenerative ‘stem-like’ properties. In this study, we characterise how the improved culture of adipose-derived mesenchymal stem cells (ASCs) within a tissue-mimetic 3D hydrogel system, that is mechanically similar to native adipose tissue, enhances their regenerative capabilities. Notably, the hydrogel system contains a porous microarchitecture that permits mass transport, enabling efficient collection of secreted cellular compounds. By utilising this 3D system, ASCs retained a significantly higher expression of ASC ‘stem-like’ markers while demonstrating a significant reduction in senescent populations, relative to 2D. Additionally, culture of ASCs within the 3D system resulted in enhanced secretory activity with significant increases in the secretion of proteinaceous factors, antioxidants and extracellular vesicles (EVs) within the conditioned media (CM) fraction. Lastly, treatment of wound healing cells, keratinocytes (KCs) and fibroblasts (FBs), with ASC-CM from the 2D and 3D systems resulted in augmented functional regenerative activity, with ASC-CM from the 3D system significantly increasing KC and FB metabolic, proliferative and migratory activity. This study demonstrates the potential beneficial role of MSC culture within a tissue-mimetic 3D hydrogel system that more closely mimics native tissue mechanics, and subsequently how the improved phenotype augments secretory activity and potential wound healing capabilities of the MSC secretome.

## 1 ∣ INTRODUCTION

Mesenchymal stem/stromal cell (MSC) therapeutics have garnered immense interest in scientific research and discovery due to the intrinsic regenerative capabilities and wide-ranging applications of MSCs.^1^ MSCs are a heterogenous population of progenitor cells easily isolated from a variety of different tissue locations, including bone-marrow, adipose, dental pulp and umbilical cord.^2,3^ MSCs maintain multilineage differentiation potential and have shown the capacity to differentiate towards a number of different end-stage mature tissue types.^3-5^ Thus, MSCs are at the forefront of tissue engineering and regenerative medical research in attempts to develop next generation bioengineered organs and tissue via combining multipotent MSC populations with bioengineered tissue scaffolds.^6-11^

MSC-based therapies have also expanded to include cell-based therapies.^12-14^ This is due to the inherent homing ability of MSCs to locations of tissue damage upon injection, in addition to the highly secretory nature of MSCs.^15-18^ Numerous studies have investigated the use of MSC therapies in both animal and human trials.^19-21^ Although the dynamics and efficacy of MSC-based therapy remains unresolved, the preliminary outcomes are potentially inspiring, so much so that over 1000 registered MSC-based clinical trials are currently listed with the FDA.^22-24^

Interestingly, recent studies have demonstrated that the clinical benefit seen in many MSC-based therapies may potentially rely less on their multipotent nature and more on their adaptive secretory response after implantation.^18,25,26^ The ability for MSCs to dynamically sense and adapt to specific environmental stimuli permits the subsequent modulation of local tissue environments in a state-dependent manner via secretion of biomodulatory factors.^27-30^ Notably, MSCs have demonstrated the capacity to secrete cytokines, growth factors and extracellular vesicles (e.g., exosomes and microvesicles) that are immunomodulatory, trophic, mitogenic, pro-angiogenic and can augment matrix deposition to promote neotissue formation.^30-34^ Therefore, MSCs contain a variety of intrinsic regenerative capabilities due to the compositional plasticity of their bioactive secretory product. Furthermore, studies have shown that wound healing and other tissue reparative processes can be enhanced after treatment solely with acellular MSC secretory products. For example, conditioned media (CM) from MSCs in 2D culture have demonstrated the capacity to directly enhance the migration, proliferation, and matrix production of fibroblasts and keratinocytes, which are key cell populations involved in the regulation of wound healing and tissue regeneration in a variety of settings.^35-39^ However, the role of a tissue-mimetic hydrogel culture system in augmenting the MSC secretory response is still under investigation. These observations have prompted a new age of MSC and MSC-derived acellular biologic therapies and adjuvants as a means to modulate the regenerative bioactivity of already utilised wound therapy modalities, in order to further promote tissue genesis and restores tissue function.^40^

Wound healing is a broad classification for the highly complex series of synchronised signalling events that occur in the setting of tissue damage.^41,42^ Two important cell populations involved with wound healing are Keratinocytes (KCs) and Fibroblasts (FBs).^43^ KCs orchestrate the process of re-epithelialization, which is a fundamental step of proper wound healing that results in ‘closure’ of the wound.^44,45^ Lack of wound closure can result in polymicrobial infections, desiccation and reinjury. Similarly, FBs are intimately involved in a number of processes during wound healing.^46^ FBs must migrate into the wound tissue, aid in the deposition of temporary granulation tissue and subsequently remodel the tissue matrix.^46^ The bioactivity of KCs and FBs is highly dependent on cellular crosstalk and communication within the local tissue environment. Thus, paracrine and autocrine signalling activity are critical to the success of native wound healing.^45,47^

In the body, our tissue microenvironments provide a range of mechanotransductive cues that regulate cellular activity within that environmental niche, including tissue mechanics.^48^ Moreover, studies have demonstrated that substrate mechanical properties play a key role in cellular differentiation, function, viability and overall phenotypic properties within in vitro culture systems.^49,50^ Notably, tissues in our body typically range from ~0.1 to 100 kPa in tissue stiffness, with the brain measuring in around ~0.3 kPa, fat ~3 kPa, muscle ~10 kPa and precalcified bone ~100 kPa.^51^ Conversely, traditional 2D tissue culture plastic is a much stiffer substrate, often reaching around ~1000 kPa or greater.^51^ Evidence suggests that traditional 2D culture is not ideal for expansion of many cells, particularly ‘stem-like’ cells such as MSCs, and can result in a loss of MSC multipotency, induction of senescence and decreased bioactivity.^52-56^ This is due to a combination of an unnaturally stiff 2D culture substrate, over-crowding of cells and the stress of continuous subculturing (i.e., passaging) to achieve adequate cell numbers for experiments and/or clinical therapies.

Cellular senescence is a progressive, phenotypically diverse and multi-staged process of cellular aging and DNA damage, typically considered to result in irreversible cell cycle arrest.^57,58^ The secretome of senescent cells, including MSCs, have been shown to negatively impact tissue regeneration and wound healing by inhibiting angiogenesis, exacerbating inflammation, increasing oxidative stress and inducing senescence in secondary cell populations.^58-61^ Thus, traditional 2D culture modalities could ultimately be resulting in less viable and ‘unhealthy’ MSC populations, leading to impurities and/or an inconsistent secretive product that subsequently limits, or possibly directly inhibits, the potential clinical benefits of MSC-derived therapeutics. Thus, current 2D expansion technologies for MSC-based therapies have created a bottleneck in developing new, effective and reproducible therapies. However, studies have shown that 3D systems can improve the ‘stemness’ and longevity of MSC populations.^62-64^ Due to this, a number of attempts have been made to generate 3D systems that are tissue-mimetic in nature, such as the fabrication of 3D hydrogels that recapitulate the native in vivo tissue mechanics cells would be exposed to.

In this study, we utilise a ‘bioinert’ 3D hydrogel system that mechanically mimics adipose tissue in order to culture Adipose-derived Mesenchymal Stem Cells (ASCs). Subsequently, the relationship between the ASC population and subsequent modulation of the ASC secretory byproducts were evaluated to determine the ability of the secretome to augment specific wound healing functions. More specifically, the loss of ‘stemness’ and induction senescence of ASCs cultured in a traditional 2D system will be compared to ASCs within the ‘bioinert’ 3D hydrogel system and the secretory byproducts of each will be used to assess for modulation of migration and proliferation of keratinocytes and fibroblasts in vitro. Utilisation of a ‘bioinert’ 3D substrate provided the opportunity to directly observe the role of the mechanical, dimensional and architectural properties of a 3D system on ASC senescence and ‘stem-like’ phenotypic properties without artificially supplementing with a biological additive or ‘bioactive’ substrate. Additionally, the 3D hydrogel system contains a porous microarchitectural design that permits mass transport and easy collection of acellular byproducts secreted from cells, essentially serving as a ‘bioreactor’. Therefore, we hypothesized that the mechanical properties of an adipose-like tissue-mimetic system would result in a more robust ASC population and subsequently alter ASC secretory activity to enhance wound healing functions in keratinocytes and fibroblasts.

## 2 ∣ MATERIALS AND METHODS

### 2.1 ∣ Cell culture

Human adipose-derived mesenchymal stem cells (ASCs; Lonza, Lot #18TL212639, 23-year-old female, Black), human keratinocytes (KCs; Lonza, Lot #18TL318559, 62-year-old male, Caucasian) and human adult dermal fibroblasts (FBs; Lifeline Cell Technologies, Lot #04057, 31-year-old female, Black) were utilised. MSC-GM (Mesenchymal Stem Cell Growth Medium) BulletKit^™^ was obtained from Lonza (Basel, Switzerland; #PT-3001) and used for ASCs. MesenCult^™^-ACF Plus Medium Kit (Stem Cell Technologies; Cat. #05445) was used as the serum-free media collected for conditioned media experiments. DermaLife K Keratinocyte Medium Complete Kit was obtained from Lifeline Cell Technologies (Maryland, USA; #LL-0007) and used for KCs. Fibro-Life Fibroblast Medium Complete Kit was obtained from Lifeline Cell Technologies (Maryland, USA; #LL-0001) and used for FBs.

### 2.2 ∣ Three-dimensional (3D) printed hydrogel cell culture system

The ‘bioinert’ 3D hydrogel system is ~1-cm^3^ and is a PEG-based 3D printed cell culture and expansion system called a Tissue-Block (T-Block; Ronawk; Kansas, USA) that contained a unique microarchitectural design with a continuous porous channelling system that had a pore diameter of 300-μm. The hydrogel is printed utilising a pre-defined and specific microstructure that results in the creation of voided/porous regions that create continuous microchannels and results in a porous fraction that account for ~42% of the total volume. The unique microarchitectural design promotes cellular migration and proliferation, while also permitting mass transport and nutrient exchange, via the microchannels. The 3D hydrogels were placed into a glass 6-well culture plate and utilised for cell culture to culture ASCs in a more tissue-mimetic environment and as a ‘bioreactor’ to generate/collect secreted byproducts from the ASCs for analysis. Fibronectin is a commonly selected coating substrate for ASCs due to their natural secretion of fibronectin. Since the cells do not naturally adhere/attach to the PEG-based (polyethylene glycol) hydrogel, both the 2D culture plastic/glass and 3D hydrogel system were coated with fibronectin 24-h before cell seeding at a standardised concentration of 5-μg/cm^2^ to enhance the initial cell attachment for experimental assays carried out in this study. The concentration of fibronectin was standardised to surface area due to the inherent surface area-to-volume differences between 2D and 3D systems. The approximate surface area of the 3D hydrogel was calculated from the 3D model used for bioprinting. After the T-Blocks were coated, ASCs were added dropwise and allowed to migrate and distribute throughout the porous microchannels. It is important to note that the cells are not embedded/encapsulated within the hydrogel itself but rather form 3D networks within the porous microchannels. Additionally, oxygen distribution within hydrogel was carried out by O2M Technologies (Chicago, IL).

### 2.3 ∣ Expansion of ASCs, KCs and FBs

ASCs, KCs and FBs were seeded on 2D plastic and cultured until ~80% confluency before subculturing (i.e., passaging). Subculturing of cells was performed by removing culture media, washing 3× with Hank’s balanced salt solution (HBSS; calcium-free, magnesium-free) and incubating with 0.05% Trypsin/EDTA (Lonza; Cat. #CC-3232) at 37°C for 5-min. Trypsin was neutralised with serum and cells were centrifuged at 500*g* for 5-min, pelleted and resuspended for reseeding and use in experimental assays or continued expansion. Subculturing events occurred every 4–5 days for ASCs, 5–7 days for KCs and 4–5 for FBs. After an initial characterisation of ‘Passage 1 (P1)’ ASCs, subcultured ASCs at ‘P2’ through ‘P5’ were utilised for the assays in this study. The increased surface area of a single 3D hydrogel system eliminated the need for subculturing for the time course of this study, therefore a passage-equivalence timepoint was utilised to allow for analogous comparison with 2D culture. For example, in this study, ASCs were seeded in 2D and 3D at ‘P2’, a passaging event occurred every 4–5 days in 2D for a total of three passages. After three passaging events in 2D ASCs became ‘P5’, thus the passage-equivalent in 3D was ‘P5’ after 14 days in 3D culture, even though the cells never underwent another passage after the initial ‘P2’ seeding. Culture expansion for 14 days was determined based on the known 2D and 3D surface areas, initial cell seeding density and average population doubling time of ~2.25 days (experimentally determined in 2D) for the ASCs in order to standardise cell numbers. ASCs were seeded at a standardized concentration of ~5000-cells/cm^2^ for assays. KCs and FBs were seeded at a concentration of ~7500-cells/cm^2^ for 2D and only grown in 2D for the experiments in this study.

### 2.4 ∣ ASC phenotype characterisation

Evaluation of ASC ‘stemness’ phenotype was performed with the initial population at ‘P1’ (Figure S1), and then again at ‘P2’ and ‘P5’ for experimental studies. ASCs at ‘P2’ were either continuously subcultured in a T-150 culture flask until ‘P5’, or seeded at a standardised density of ~5000-cells/cm^2^ onto a 96-well glass bottom 2D culture plate (Cellvis; Cat. #P96-1.5H-N) or within the 3D hydrogel system for assessment. ASCs in the 96-well plate were allowed to culture in serum-based media for 2 days, fixed, then assessed for ASC phenotype via immunolabelling of surface CD markers, ASC ‘stem-like’ phenotype is approximated by exhibiting positive staining for CD73, CD90 and CD105 and negative staining for CD34 and CD45. After fixation with 4% paraformaldehyde, cells were washed 3× with HBSS and then placed in blocking buffer (1% donkey-serum in HBSS) for 1-h. After blocking, primary antibodies for CD73 (Abcam; Cat. #133582; 1:100), CD90 (Abcam; Cat. #181469; 1:100), CD105 (Abcam; Cat. #231774; 1:100), CD34 (Abcam; Cat. #81289; 1:200) or CD45 (Abcam; Cat. #40763; 1:200) were applied to the appropriate wells and allowed to incubate overnight at 4°C. The next day cells were washed 3× with blocking buffer followed by application of secondary antibodies for 1-h, then 3× washes with HBSS again. Cells were counterstained with an immunofluorescent nuclear marker, Hoechst 33342 (Invitrogen; Cat. #H3570; 1:1000) and Alexa Fluor 647 Phalloidin (Invitrogen; Cat. #A22287; 1:1000). Immunofluorescence was assessed with a Revolve microscope (Echo, San Diego, CA, USA; DAPI: EX-380/30, EM-450/50; FITC: EX-470/40, EM-525/50; TXRED: EX-560/40, EM-630/75; CY5: EX-630/40, EM-700/75) and 20× objective (Olympus; UPlanSApo, 0.75NA). ASC ‘stem-like’ phenotypic characterisation was carried out in biological quadruplicate (*n* = 4), with a total of up to ten (10) field of view images taken per biological replicate (2D = per well, 3D = per hydrogel), to achieve up to forty (40) total measured values for each group. The ten (10) technical replicates were averaged for each replicate for statistical analysis. Total nuclei were counted, and total positive cells were evaluated. Phalloidin counterstain was used to aid in localization of positive CD marker staining. The ‘P2’ ASCs were used as an experimental baseline measurement of ‘stemlike’ phenotype. ASCs were continuously expanded in 2D until ‘P5’. Upon cells reaching ‘P5’ (14 days), cells were assessed for ‘stem-like’ phenotype, as mentioned above. ASCs seeded in 3D system at ‘P2’ were assessed simultaneously at the ‘P5’ passage-equivalent timepoint for 3D.

### 2.5 ∣ Analysis of ASC proliferation and migration within porous hydrogel system

The ability for ASCs to adhere to, and propagate within, the hydrogel system was evaluated via confocal microscopy after prolonged culture based on similarly discussed protocols. In short, ASCs were seeded onto/within the hydrogel system at a standard seeding concentration. MSC-GM media was changed every two (2) days for two (2) weeks (14 days). At the conclusion of the culture expansion, the ASC-hydrogel was washed 3× with HBSS for 5 min each, fixed with 4% paraformaldehyde, and stained. Staining protocols included Hoechst 33342 (Invitrogen; Cat. #H3570; 1:1000) and Alexa Fluor 488 Phalloidin (Invitrogen; Cat. # A12379; 1:500), and MitoTracker^™^ Red CMXRos (Invitrogen; Cat #M7512; 500 nM), and were carried out per the manufacturer’s recommended protocol. Immunofluorescence of ASCs within the hydrogel system was assessed with a Leica TCS SPE Laser Scanning confocal equipped with a 405 nm, 561 nm and 635 nm lasers and a 10× objective (Leica; ACS APO, 0.3NA).

### 2.6 ∣ ASC senescence characterisation

Similar to ASC phenotyping methodology in Section 2.4, assessment of ASC senescence was performed. ASCs at ‘P2’ were either continuously subcultured in a T-150 culture flask until ‘P5’, or seeded at a standardised density of ~5000-cells/cm^2^ onto a 96-well glass bottom 2D culture plate (Cellvis; Cat. #P96-1.5H-N) or within the 3D hydrogel system for assessment. ASCs in the 96-well plate were allowed to culture in serum-based media for 2 days, fixed, then assessed for senescent activity via immunofluorescent labelling of β-galactosidase activity with the CellEvent^™^ Senescence Green Detection Kit (Invitrogen; Cat. #C10850), per manufacturer’s instructions. The ‘P2’ ASCs were used as a baseline measurement of senescence. ASCs were continuously expanded in 2D until ‘P5’. Upon cells reaching ‘P5’ (14 days), cells were again seeded onto glass well plate and assessed for senescent activity, as mentioned above. ASCs initially seeded in 3D system at ‘P2’ were assessed simultaneously at the ‘P5’ passage-equivalent timepoint for 3D. Cells were counterstained with an immunofluorescent nuclear marker, Hoechst 33342 (Invitrogen; Cat. #H3570; 1:1000) and Alexa Fluor 647 Phalloidin (Invitrogen; Cat. #A22287; 1:1000). Immunofluorescent was assessed with a Revolve microscope (Echo, San Diego, CA, USA; DAPI: EX-380/30, EM-450/50; FITC: EX-470/40, EM-525/50; TXRED: EX-560/40, EM-630/75; CY5: EX-630/40, EM-700/75) and 20× objective (Olympus, UPlanSApo, 0.75NA). Senescence characterisation was carried out in quadruplicate (*n* = 4), with a total of ten (10) field of view images taken per biological replicate (2D = per well, 3D = per hydrogel), to achieve forty (40) total measured values for each sample. The ten (10) technical replicates were averaged for each replicate for statistical analysis. Total nuclei were counted, and total senescent positive and negative cells were counted. The percent (%) ‘Senescent Positive’ was evaluated. Phalloidin counterstain was used to aid in localization of positive senescent marker staining.

### 2.7 ∣ ASC gene expression

RNA was isolated and purified via an RNeasy Mini Kit (Qiagen), according to manufacturer’s instructions, for 2D and 3D samples at ‘P5’ in addition to 2D samples at ‘P2’ which served as a baseline expression control. Only RNA with a 260/280 ratio of >1.8 were used for this study. Samples were reversed transcribed into cDNA using a RT^2^ First Strand Kit (Qiagen; Cat. #330404) and a qTower3 Real-Time Thermocycler (Analytik Jena), according to manufacturer’s instructions. Purity of cDNA samples was assessed with a QuickDrop spectrophotometer (Molecular Devices), with a 260/280 absorbance ratio > 1.8 was designated as pure. Gene expression was assessed using real-time quantitative polymerase chain reaction (RT-qPCR) using a qTower3 real-time thermocycler. A Qiagen RT^2^ Profiler^™^ PCR Array for Human Mesenchymal Stem Cells (PAHS-082ZC-24) was used to assess for genomic expression of 84 MSC and MSC-associated genes. Cycle threshold (Ct) values were recorded and analysed via the Delta–Delta-Ct method. Glyceraldehyde 3-phosphate dehydrogenase (GAPDH), Beta-actin (ACTB) and Beta-2-Microglobulin (B2M) were the endogenous control genes utilised by the array. The entire list of 84 genes and relative fold changes for 2D and 3D can be found in Table S1. Additionally, individual qPCR primers (Qiagen; Cat. #330001) were purchased to perform RT-qPCR on p16 (*CDKN2a/INK4a*; Gene Globe ID: PPH00150F-200) and p53 (Gene Globe ID: PPH00213F-200) to directly assess gene expression of senescence-associated genes. GAPDH was used as an endogenous control for these samples as well. Reverse transcription and gene expression analysis was performed as described above and all RNA analyses were performed with biological triplicates (*n* = 3).

### 2.8 ∣ Isolation of ASC conditioned media

Media supplementation was standardised to ~250-μL/cm^2^ for ASC expansion to account for dilutional differences in surface area-to-volume ratio between 2D and 3D culture. Media was changed every 2 days. When Conditioned Medium (CM) from ASC culture was desired, MSC-GM media was removed, cells were washed with HBSS and serum-free MSC media was added for 48-h before collection for both 2D and 3D. Collected ASC-CM was then centrifuged at 1500*g* for 10-min to eliminate cell debris, Steriflip filtered with a 0.22-μm filter and stored at −80°C for long-term storage until use.

### 2.9 ∣ Proteomic microarray for secreted soluble proteins

ASC conditioned media (ASC-CM) was collected from ‘P3’ cells in 2D, or ‘P3’ passage-equivalent cells in 3D that were originally seeded at ‘P2’. Media was collected per previous protocol, via centrifugation and filtration prior to use in proteome array. A Proteome Profiler Human Angiogenesis Array Kit (R&D Systems, Inc., Minneapolis, MN, USA; Cat. #ARY007) was carried out according to the manufacturer’s instructions. The array allows for chemiluminescent detection of 55 human proteins secreted within the ASC-CM that are related to, but not exclusive to, angiogenesis. For a full list see Table S2. Without an internal reference control on the proteome array blots, 2D and 3D samples were performed and developed pairwise to standardise exposure time for each pair. For example, analysis of one (1) 2D blot and one (1) 3D blot array was performed in tandem and placed into chemiluminescent detector machine (ProteinSimple, Inc., San Jose, CA, USA; FluorChem E) at same time and exposed simultaneously. The relative fold change of 3D-to-2D signal was calculated for each pair of 2D and 3D samples using ImageJ software and performed in biological triplicate (*n* = 3). The average fold change was then calculated.

### 2.10 ∣ ASC-CM extracellular vesicle (EV) production

ASC-CM was collected as previously discussed and total EV production was assessed via relative protein content of the EV fraction. EVs were isolated via centrifugation at 3500*g* for 15-min through a 100-kDa filter tube (Cytiva; Cat. #28932363) followed by washing with PBS and re-centrifugation at 3500*g* for 5-min through the 100-kDa filter, for a total of 3 washes. The concentrate was then taken, and EV were precipitated overnight using a ExoQuick-TC kit (SBI; Cat. # EXOTC10A-1), per the manufacturers protocol. In short, samples were centrifuged again 1500*g* for 30-min and the supernatant was removed. A second centrifugation 1500*g* for 5-min was performed to remove residual solution. EV were resuspended in 500-μL of PBS. Samples were then used for downstream analysis via protein quantification with a Pierce^™^ BCA Protein Assay Kit (Invitrogen; Cat. #23225), Pierce^™^ Coomassie ‘Bradford’ Protein Assay Kit (Invitrogen; Cat. # 23200), and a QuickDrop (Molecular Devices; SpectraMax QuickDrop Micro-Volume Spectrophotometer) quantification via absorbance at 280-nm. Relative protein content within the EV fraction was determined and back-calculated to determine what the relative concentration was within media before 100-kDa concentration (e.g., 10-mL of media concentrated to 500-μL was a 20× concentration). Assays were performed with technical replicates and biological triplicates (*n* = 3). Additionally, purified EV/exosome samples were evaluated via Nanoparticle Tracking Analysis (NTA; Malvern Panalytical; Nanosight LM10) to determine both concentration and size distribution of particles. NTA analysis was performed with three biological replicates (*n* = 3) and five technical replicates for each biological replicate.

### 2.11 ∣ Keratinocyte and fibroblast activity after ASC-CM treatment

ASC-CM was collected from ‘P5’ cells in 2D, or ‘P5’ passage-equivalent cells in 3D that were originally seeded at ‘P2’. Media was collected per previous protocol, via centrifugation and filtration prior to use in proteome array. ASC-CM was then placed on Keratinocytes (KCs) for up to 24-h and Fibroblast (FBs) for up to 32-h to assess for capacity to modulate metabolic, proliferative or migratory activity. Standardised seeding density of KCs and FBs from ‘P1’ with KC Growth Media (KC-GM) or FB Growth Media (FB-GM) was performed and incubated for 24-h to allow for cells to attach and acclimate before ASC-CM treatment. After 24-h, the media was removed, cells were washed with HBSS, ASC-CM was applied and experimental assays were performed per the manufacturer’s instructions. PrestoBlue fluorescence was obtained at 560/590 nm (*n* = 4) and used for evaluation of metabolic activity after 24-h of ASC-CM culture. Hoechst 33342 was added to PrestoBlue samples and values were displayed as an average relative fluorescent unit (R.F.U.) of PrestoBlue per Hoechst signal in order to control for potential differences in cell numbers and obtain approximate metabolic activity per cell. PrestoBlue results were then compared relative to treatment with control media only (KC-GM or FB-GM) for both 2D and 3D ASC-CM and between 2D versus 3D treatment. PicoGreen fluorescence was obtained at 485/535 nm (*n* = 4) and used for evaluation of cell number as a surrogate measurement of KC and FB proliferation after 24-h of ASC-CM culture. Total cell numbers per 96-well were calculated for PicoGreen based on DNA content and an average DNA content per cell being 7.7-pg/cell. KC and FB scratch assays were performed to evaluate changes in migratory activity via changes in area after scratching with a pipette tip to generate a voided space (*n* = 3). Migration images were taken every 4-h using an ImageXpress Micro XLS Imaging System (Molecular Devices). The entire area was imaged for each triplicate and three (3) different regions per triplicate were used to calculate change in area. The three (3) area values were averaged per triplicate and per time point for each group and displayed as percent (%) area recovered as a surrogate measurement of migration (*n* = 3).

### 2.12 ∣ EV in vitro tracking

EVs previously isolated from the ASC-CM were fluorescently labelled with the lipophilic membrane stain Dil (Invitrogen; Cat. #D282) at a concentration of 1-μM. The labelled EVs were washed with PBS and re-centrifuged at 3500*g* for 5-min through the 100-kDa filter, for a total of 3 washes and centrifugations to remove excess dye. DiI-labelled EVs were added to KC-GM or FB-GM and co-cultured overnight at 37°C with KCs and FBs, respectively. The following day, the cells were washed 2× with PBS and then fixed with 4% paraformaldehyde, followed by 2× additional PBS washes. KCs and FBs were counterstained with Hoechst 33342 (Invitrogen; Cat. #H3570; 1:1000) and Alexa Fluor 488 Phalloidin (Invitrogen; Cat. #A12379; 1:500) and observed under a fluorescence microscope.

### 2.13 ∣ Statistical analysis

All data are reported as means with standard deviation. Characterisation analyses of ASC population with immunolabelling for senescence were evaluated with a two-way ANOVA to include control population. Immunolabelling for CD markers was also evaluated with a two-way ANOVA. Proteome array and KC scratch assay were also evaluated with a two-way ANOVA methodology. Analysis of antioxidant activity, EV protein content, and plate reader spectroscopy data for KC metabolic and proliferative activity were evaluated with a oneway ANOVA. Data was tested for normality via Shapiro–Wilk and Kolmogorov–Smirnov tests and plotted with a QQ plot. GraphPad Prism 9.0.2 software (La Jolla, CA) was used for the analyses and a *p* < 0.05 was considered significant.

## 3 ∣ RESULTS

### 3.1 ∣ Culture of ASCs within adipose-like porous hydrogel system

**FIGURE 1:**
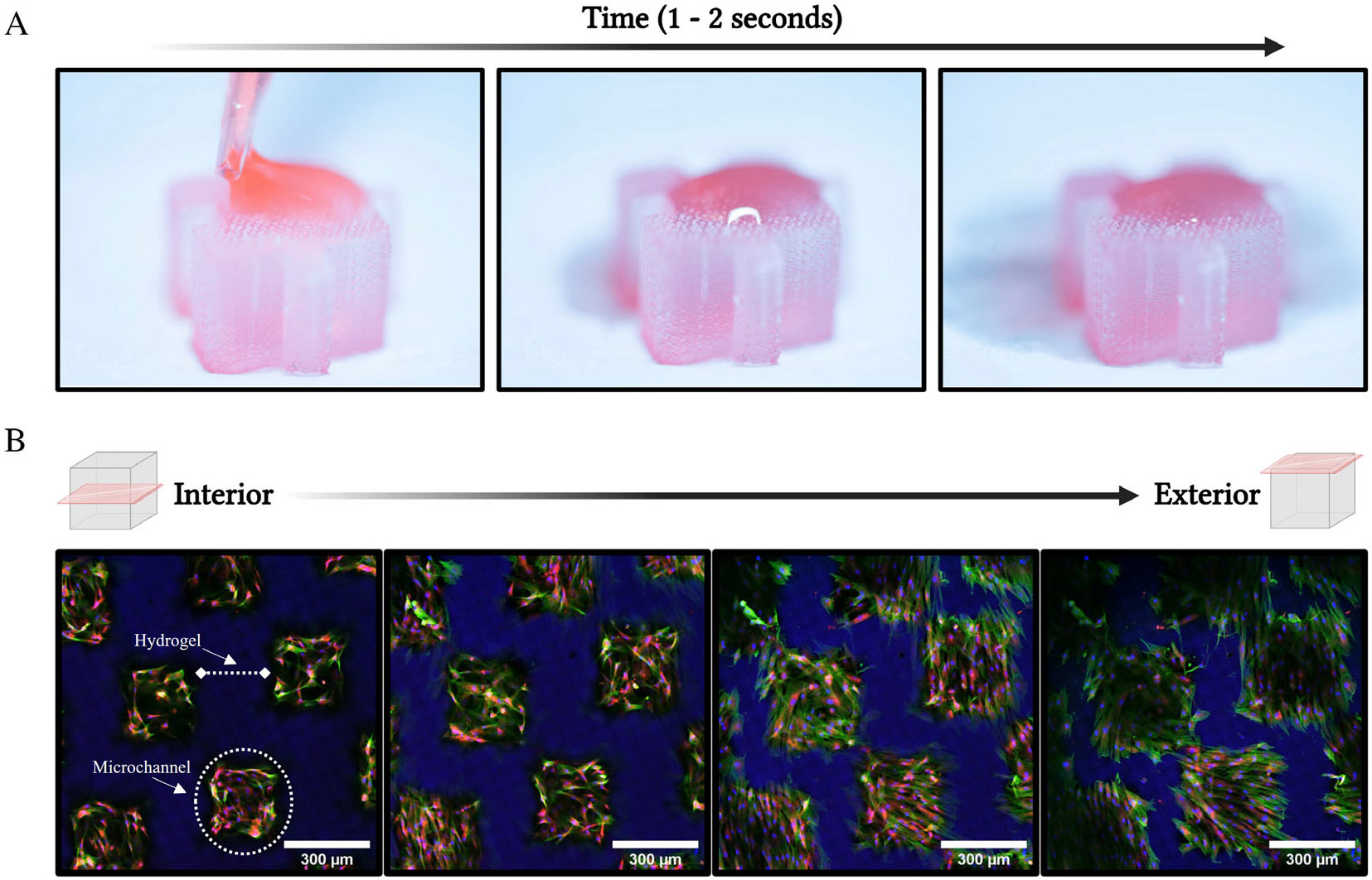
Culture of ASCs within porous hydrogel system. (A) Demonstration of fluid transport through 3D hydrogel system via application of liquid media to superficial surface of hydrogel. Sequential imaging of the system was taken as the fluid migrated through the pores of the hydrogel. Progression of time moves from leftmost image to rightmost image. Total elapsed time was ~1 to 2 s. Bottom of hydrogel set on white Kimwipe which demonstrates absorption of fluid as it migrates through the hydrogel. (B) Confocal image stacks shown sequentially with section located deepest within the hydrogel shown first (leftmost) and the most superficial section shown last (rightmost). ASCs seeded at ‘P2’ for 2 weeks. ASCs are seen populating within the porous architecture of the 3D tissue-mimetic hydrogel rather than embedded within the hydrogel. Stains include Hoechst 33342 (Blue), Phalloidin-AF488 (Green) and MitoTracker (Red). Scale bar = 300 μm.

The movement of fluid through the hydrogel porous microarchitecture was captured photographically and can be seen quickly (1–2 s) permeating from the surface of the hydrogel to the base, where the liquid was absorbed via a Kimwipe (Figure 1A). Similarly, the ability of nutrients and gases to distribute throughout the 3D system was indirectly assessed via a conjugated oxygen isotope and magnetic resonance, which demonstrated relatively homogenous distribution throughout the hydrogel (Figure S2). Additionally, ASCs were cultured within the hydrogel system for 14 days and allowed to migrate throughout the hydrogel within the porous microchannels. ASCs were then stained and assessed with confocal microscopy for their ability to distribute, via migration and proliferation, throughout the hydrogel’s pore system (Figure 1B). ASCs were seen lining the porous channel beyond the superficial surface, deep into the hydrogel system and can be seen forming 3D cellular networks within the hydrogel’s pore structures. Moreover, there is no indication of cell death or necrosis within the more centralised region of the hydrogel.

The mechanical properties of the hydrogel were characterised to assess for similarities with native adipose tissue. Notably, based on literature, the modulus (E) of adipose tissue is about 1-10 kPa when evaluated via a stress–strain curve immediately preceding plastic deformation,^65,66^ with an average considered to be ~3 kPa.^51^ DMA was performed to determine the elastic compressive secant modulus (E), max strain and max stress of the hydrogel system. The modulus (E) was determined to be ~3.7 kPa immediately prior to plastic deformation (Figure S3), similar to what has been reported for native adipose tissue.

### 3.2 ∣ 3D hydrogel expansion decreases ASC senescence

**FIGURE 2:**
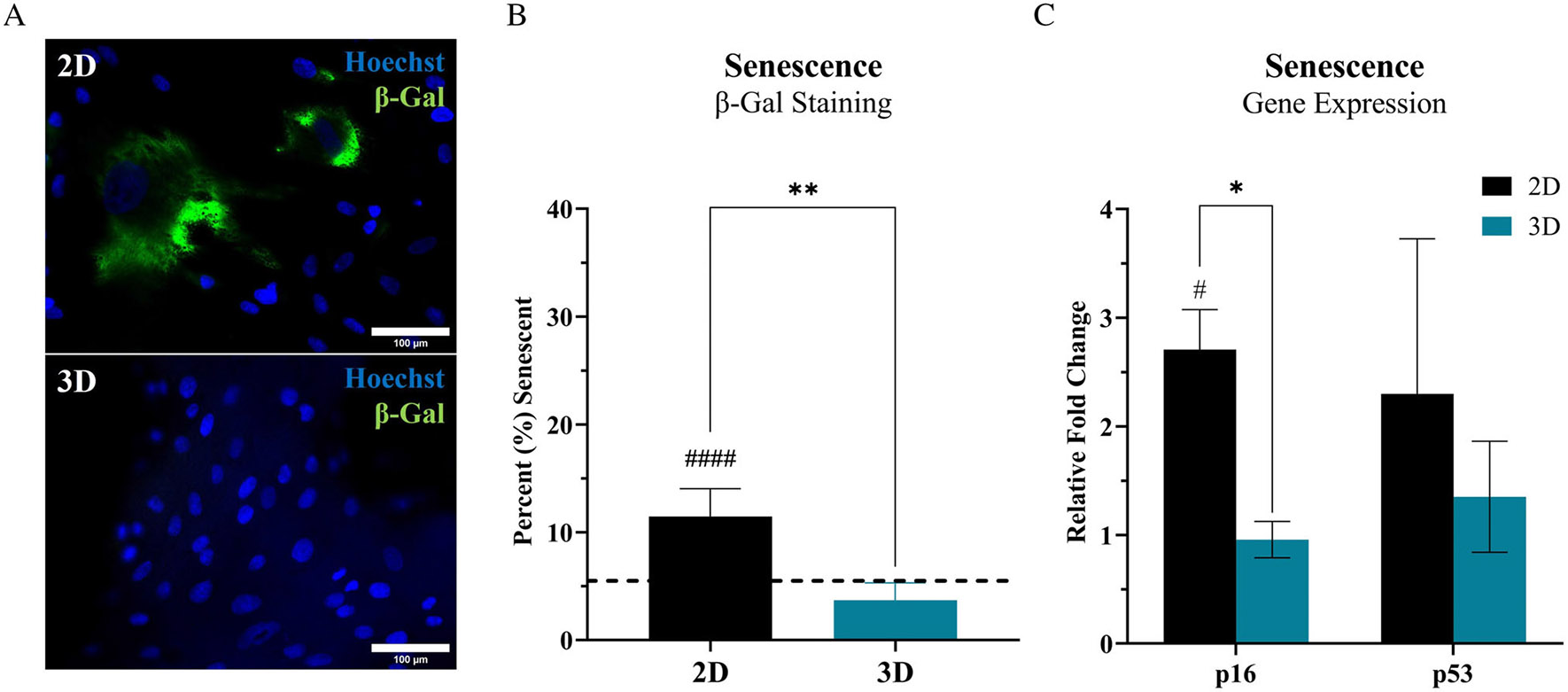
Tissue-mimetic hydrogel culture decreases ASC senescence. ASCs seeded at ‘P2’ within the 3D hydrogel system or continuously subcultured for 2 weeks in traditional 2D culture until reaching ‘P5’. The ‘P5’ ASCs were used for characterisation in 2D and ‘P5’ passage-equivalent were used for 3D. (A) At the conclusion of culture period, ASCs were fixed and stained for senescence/β-galactosidase (Green), Hoechst 33342 (Blue) and Phalloidin-AF647 (Not Shown). (B) ASCs cultured in 2D (Black Bar) or 3D (Teal Bar) were evaluated using a 20× objective. ASCs seeded in 2D at ‘P2’ were used as an initial control population and denoted as the dashed line (Black). All image quantification data is displayed as a bar graph and is the result of averaging each group of technical replicates (different images within each biological replicate) to quantify senescence. Samples done is quadruplicate (*n* = 4). Scale bar = 100 μm. (C) Relative fold change in gene expression for ‘P5’ ASCs in both 2D and 3D was assessed for changes in senescence-associated markers, p16 (left) and p53 (right), relative to ‘P2’ baseline control cells. Samples done in triplicate (*n* = 3). All error bars are standard deviation. One-way ANOVA with Tukey’s post-hoc was used for statistical analysis. Significance is denoted as *p < 0.05 or ****p < 0.0001 for 2D versus 3D comparison and ^#^*p* < 0.05 or ^####^*p* < 0.0001 for comparison relative to ‘P2’ control.

ASC senescence was assessed via a marker targeting β-galactosidase activity (Figure 2A,B), a commonly utilised surrogate measurement of cellular senescence and expression of p16 and p53 (Figure 2C). Image quantification demonstrated a significant increase in senescent-positive cells at ‘P5’ within the 2D culture system (~11.5%) relative to ‘P5’ cells within the 3D culture system (~3.6%) and the initial ‘P2’ ASC population (~5.5%) (Figure 2B). Therefore, there was an ~3.2× fold increase in senescent ASCs within 2D culture relative to 3D culture. Gene expression for senescent ASC populations was assessed via p16 and p53 (Figure 2C). The relative fold change in p16 expression for ‘P5’ ASCs in 2D (~2.7×) was significantly increased relative to 3D (~1×) and to the ‘P2’ ASC baseline population. Whereas expression of p53 in 2D (~2.3×) demonstrated an increasing trend but was not significantly different relative to ASCs in 3D (~1.3×) or ‘P2’ controls. ASCs demonstrated no significant changes in senescence activity for cells cultured within the 3D system relative to ‘P2’ baseline ASCs.

### 3.3 ∣ 3D hydrogel expansion improves retainment of ASC phenotype

**FIGURE 3:**
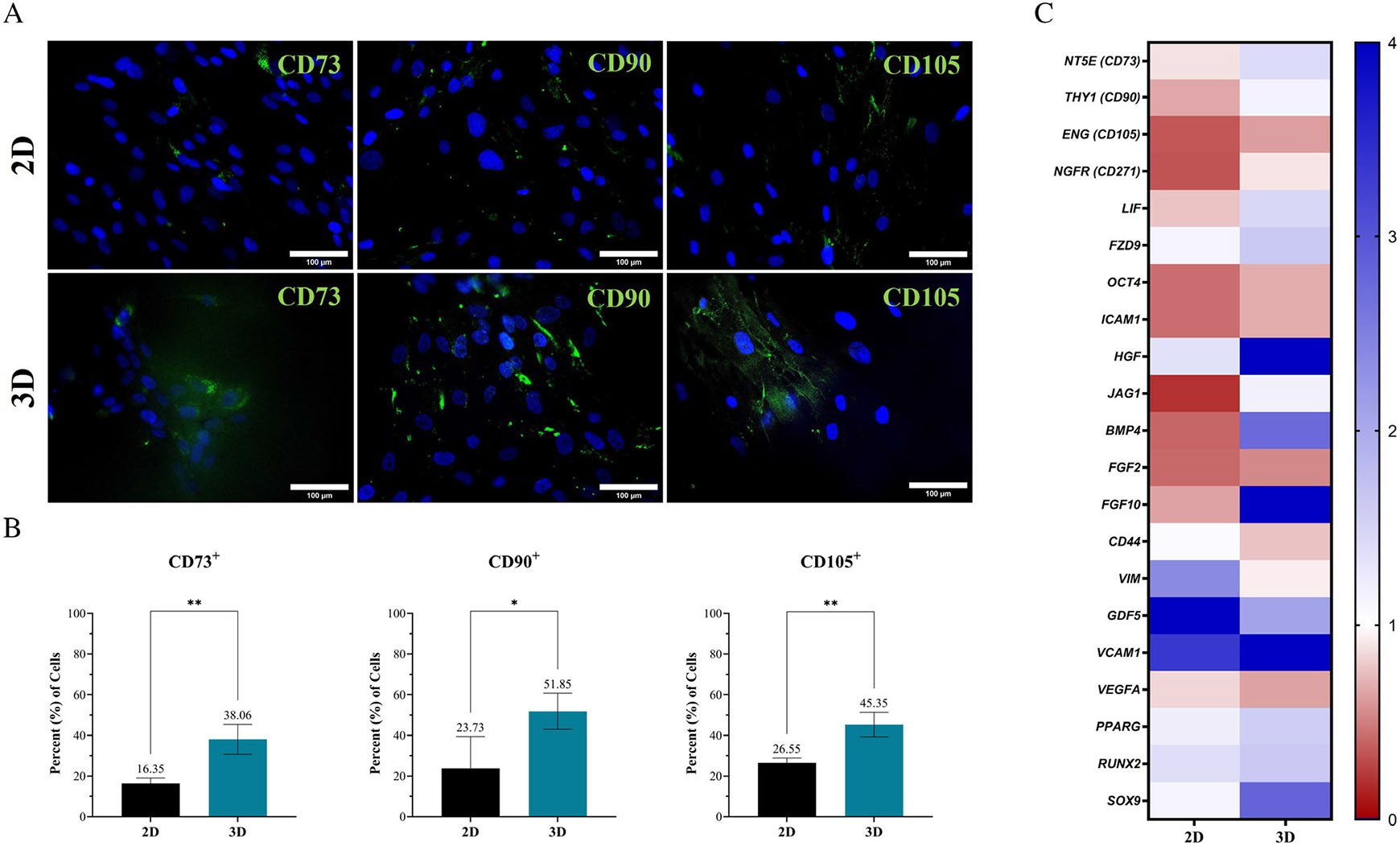
Tissue-mimetic hydrogel culture improves retainment of ASC phenotype. ASCs seeded at ‘P2’ within the 3D hydrogel system of continuously subcultured for 2 weeks in traditional 2D culture until reaching ‘P5’. The ‘P5’ ASCs were used for characterisation in 2D and ‘P5’ passage-equivalent were used for 3D. (A) At the conclusion of culture period, ASCs were fixed and stained for either CD73, CD90 or CD105 (Green) and CD34 or CD45 (Not Shown). Samples were counterstained with Hoechst 33342 (Blue). Representative images of 2D (Top Row) and 3D (Bottom Row) samples. (B) Quantification of imaging data performed and total percent (%) positive cells denoted with bar graphs for each marker (Bottom Panel). ASCs in 2D (Black Bar) or 3D (Teal Bar) were evaluated using a 20× objective. Samples done is quadruplicate (*n* = 4). Scale bar = 100 μm. Error bars are standard deviation. One-way ANOVA with Tukey's post-hoc used for statistical analysis. Significance is denoted as ****p < 0.0001. (C) A heatmap representing the relative fold change of twenty-one (21) key genes are displayed for the gene expression of ‘P5’ ASCs in 2D (left) or 3D (right). Fold change is relative to baseline control ‘P2’ ASCs (indicated by white colour) Downregulation of gene expression denoted with ‘red’ colour and up-regulating denoted with ‘blue’ colour. Values are normalised to a group of endogenous control genes that included GAPDH, ACTB and B2M.

Evaluation of ‘stem-like’ ASC populations was assessed via immunolabel quantification of cell surface CD markers (Figure 3A,B) and expression of key phenotypic markers (Figure 3C and Figure S4). Both 2D and 3D resulted in a decrease in ‘stem-like’ positive markers (CD73/90/105) over time, relative to the initial population (Figure 3B and Figure S1). ASCs exhibited 16%, 30% and 27% in 2D and 38%, 52% and 45% positive expression for CD73, CD90 and CD105, respectively. Thus, the relative decline in ASC surface markers was greater in 2D (Figure 3B). Both 2D and 3D ASCs maintained a relatively low population (<5%) of ASCs staining positive for both CD34 and CD45 throughout.

Relative gene expression of MSC and MSC-associated ‘stem-like’ genes were evaluated with an MSC phenotyping array (Figure 3C). Expression of genes for ‘P5’ ASCs in 2D or 3D are compared relative to baseline ‘P2’ ASC expression. Similar to the image quantification data, the gene expression for CD73 (NT5E; 5′-Nucleotidase Ecto), CD90 (THY1; Thymocyte Differentiation Antigen 1) and CD105 (ENG; Endoglin) demonstrated a larger decrease in 2D. Also, CD271 (NFGR; Nerve Growth Factor Receptor), another common MSC marker, exhibited a decreased expression in 2D (Figure 3C). Other MSC-associated genes, including LIF (Leukemia Inhibitory Factor), JAG1 (Jagged1), FGF10 (Fibroblast Growth Factor-10) and HGF (hepatocyte Growth Factor) exhibited significant decreases in expression for 2D culture, relative to 3D ASCs. A similar decreasing trend was observed for number of other ‘stemness’ genes including the expression of FZD9 (Frizzled-9), OCT4 (Octamer-binding transcription factor 4), FGF2 (Fibroblast growth Factor-2) and ICAM1 (Intercellular Adhesion Molecule 1) for ASCs in 2D (Figure 3C). Table S1 display values for all genes included in the array.

### 3.4 ∣ 3D hydrogel culture results in altered ASC secretory activity

**FIGURE 4:**
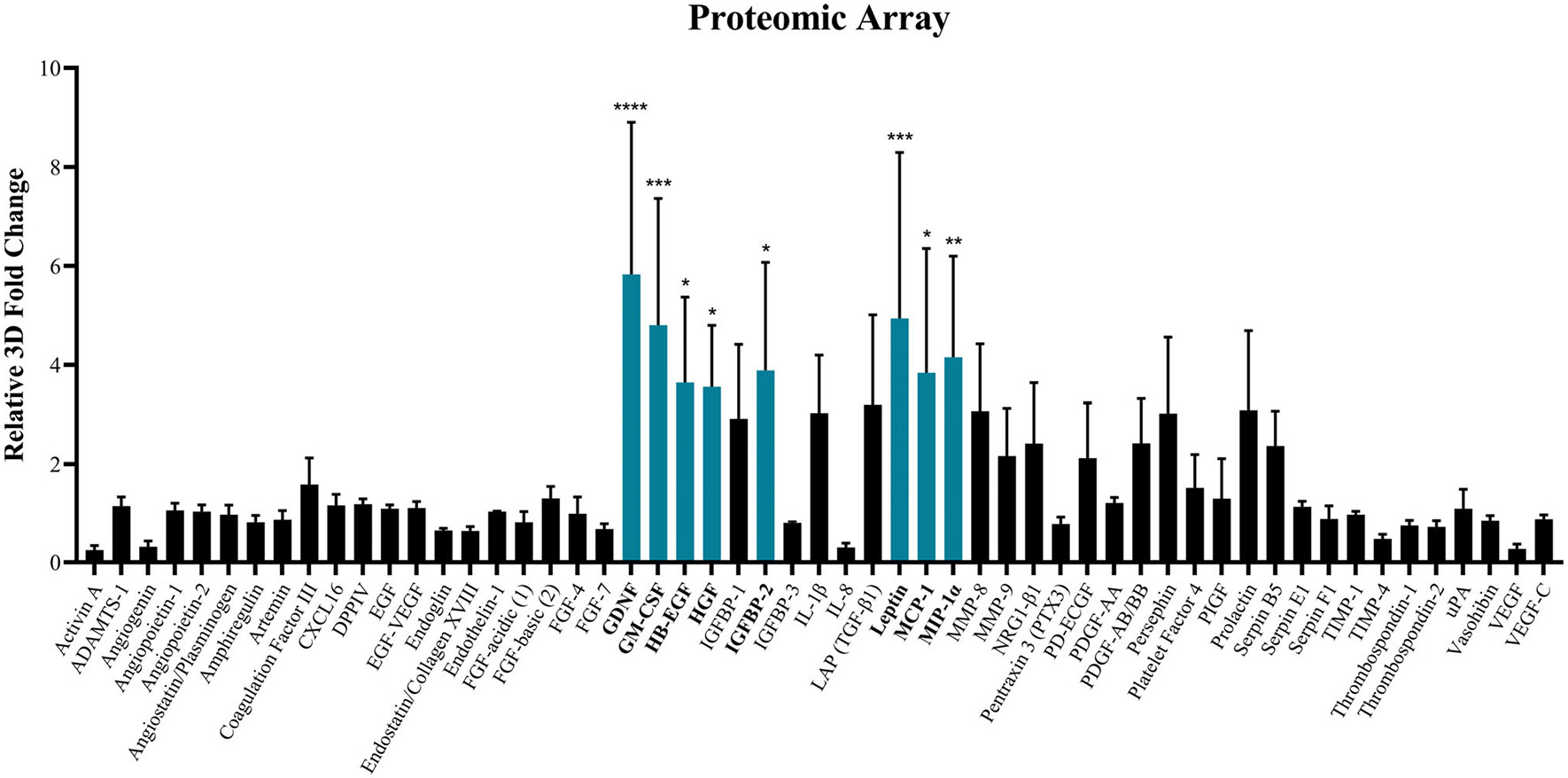
Altered secretory activity of ASCs within tissue-mimetic hydrogel. ASCs seeded at ‘P2’ within the 3D hydrogel system or subcultured one additional time in traditional 2D culture until reaching ‘P3’. The ‘P3’ ASCs were used for characterisation in 2D and ‘P3’ passage-equivalent were used for 3D. ASC-CM was collected from the ‘P3’ and ‘P3’ passage-equivalent ASC cultures, for 2D and 3D, respectively. Relative chemiluminescence was determined for each proteome array membrane. Average fold change was calculated and displayed as 3D:2D ratio. Assay was performed in triplicate (*n* = 3) and averaged. Significant differences in 3D relative to 2D are denoted (Teal Bars). Proteins indicating no significant difference are denoted with black bars. Error bars are standard deviation. One-way ANOVA with Tukey's post-hoc used for statistical analysis. Significance is denoted as *p < 0.05, **p < 0.001, ***p < 0.001 and ****p < 0.0001 for 2D versus 3D comparison.

ASC secretive bioactivity was evaluated to determine if the improved retainment of non-senescent, ‘stem-like’ ASC populations within the 3D system resulted in augmented secretory activity and altered ASC-CM. A proteomic microarray was first performed to screen for relative compositional differences between ASC-CM collected from 2D and 3D culture (Figure 4). Of the 55 total secreted proteins assessed, 8/55 (15%) were significantly increased in 3D culture relative to 2D culture, whereas the remaining 47 indicated no significant differences (Figure 4). Factors increased in 3D culture encompassed both cytokines and growth factors compounds important for the wound healing response. Additionally, the ASC-CM from 2D and 3D was evaluated for differences in total antioxidant activity (Figure S5). ASC-CM from 3D culture demonstrated a significant increase in total antioxidant activity of ~25%, relative to 2D (0.21 mM vs. 0.17 mM UA equivalents) (Figure S5).

### 3.5 ∣ 3D hydrogel enhances ASC EV production

**FIGURE 5:**
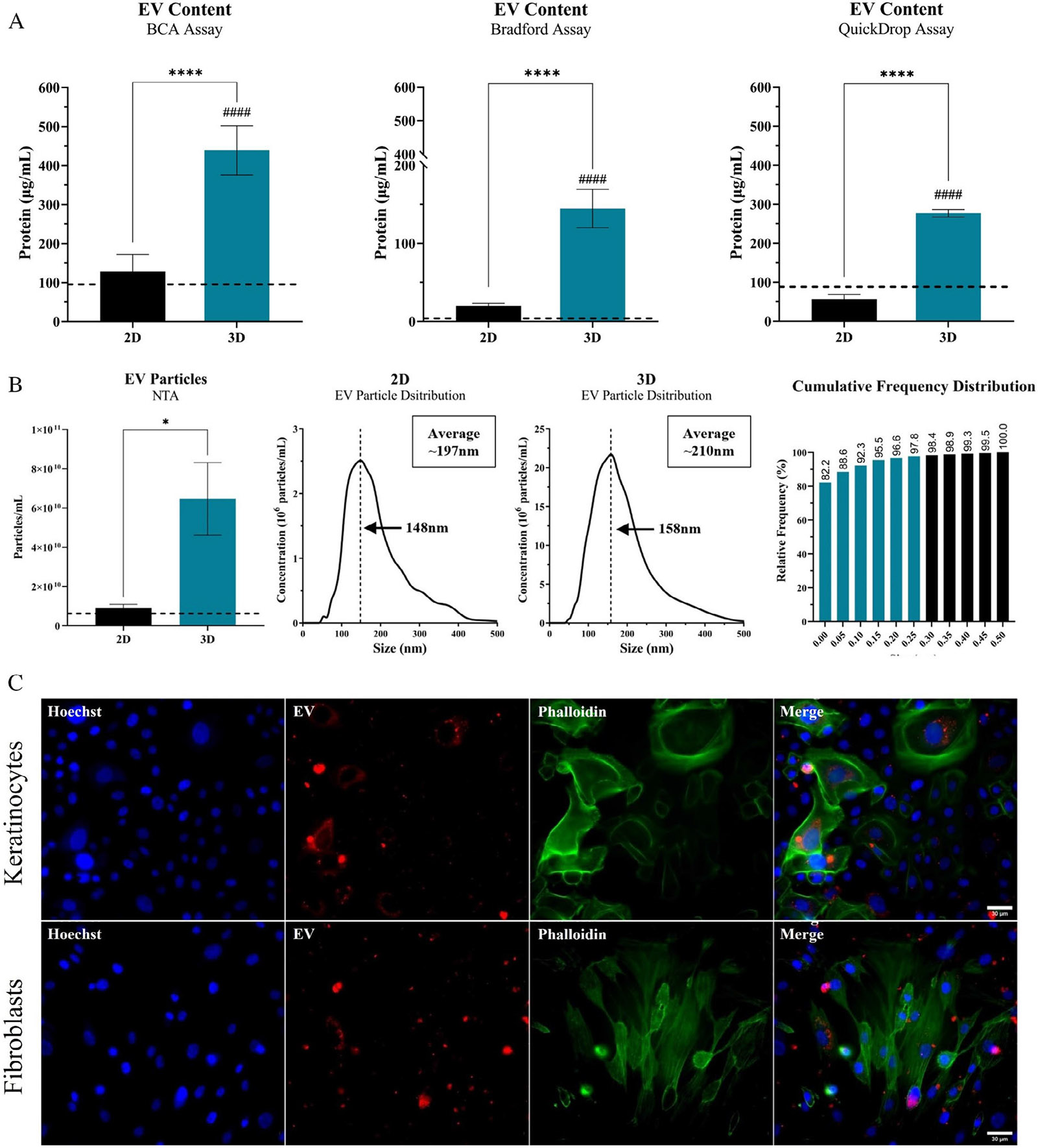
Enhanced production of EVs within tissue-mimetic hydrogel. (A) ASCs seeded at ‘P2’ within the 3D hydrogel system or continuously subcultured for 2 weeks in traditional 2D culture until reaching ‘P5’. The ‘P5’ ASCs were used for characterisation in 2D and ‘P5’ passage-equivalent were used for 3D. ASC-CM was collected from the ‘P5’ and ‘P5’ passage-equivalent ASC cultures. The EV fraction of ASC-CM was isolated, purified and analysed for relative protein content via three different modalities, BCA (leftmost), Bradford (middle) and QuickDrop (rightmost). Concentration of EV protein fraction displayed as average ‘μg/mL’ within the initial cell culture volume before concentrating with 100-kDa filter. Concentration within control media was analysed and is displayed as dashed line (Black). (B) Isolated EV fractions were then analysed with NTA for determining concentration of particles/mL within media (leftmost) and to assess size distribution of the measured particles and the cumulative frequency of the different EV particle sizes (rightmost) to determine whether measure particles are truly within EV size range. Assays were performed in triplicate (*n* = 3) and averaged. Error bars are standard deviation. One-way ANOVA with Tukey's post-hoc used for statistical analysis. Significance denoted as ****p < 0.0001 for 2D versus 3D comparison and ^####^*p* < 0.0001 for 3D comparison relative to media control. (C) Representative images of fluorescent-labelled Keratinocytes (Top) and Fibroblasts (Bottom) that were treated with media supplemented with labelled-EVs from 3D ASC-CM for 18 h. Samples imaged with a 40× objective. Scale bar = 30 μm.

ASC production of EVs was quantified within the ASC-CM from 2D and 3D culture to determine relative differences in EV composition. All three protein quantification modalities (BCA, Bradford, QuickDrop) demonstrated similar trends with a significant increase in EV protein content within the ASC-CM from 3D culture (Figure 5A). The purified EV fraction was also assessed via NTA to further determine particle concentration and size distribution between 2D and 3D EVs. NTA determined a similar trend in EV production for 3D, with approximately 9.2 × 10^9^ particles/mL in 2D and 6.5 × 10^10^ particles/mL in 3D (Figure 5B). The EV particle size distribution between 2D and 3D both had similar trends and followed a Gaussian distribution, with ~98% of all particles measured falling within the exosomal range of 25–250 nm in diameter (Figure 5B), suggesting that the EV population is likely mostly exosomal in nature. Subsequently, an in vitro tracking and uptake study was performed with the EV fraction, where the EVs were stained with a lipophilic membrane dye (DiI) and KCs and FBs were treated with the DiI-labelled EVs (Figure 5C). The EVs can be seen being taken up and endocytosed by both the KCs and the FBs within 18 h (Figure 5C). Interestingly, KC morphology and cytoskeletal rearrangement was evident (denoted by changes in phalloidin staining), especially in KCs taking up a larger proportion of EVs (Figure S6).

### 3.6 ∣ Secretome from 3D culture enhances KC and fibroblast activity

**FIGURE 6:**
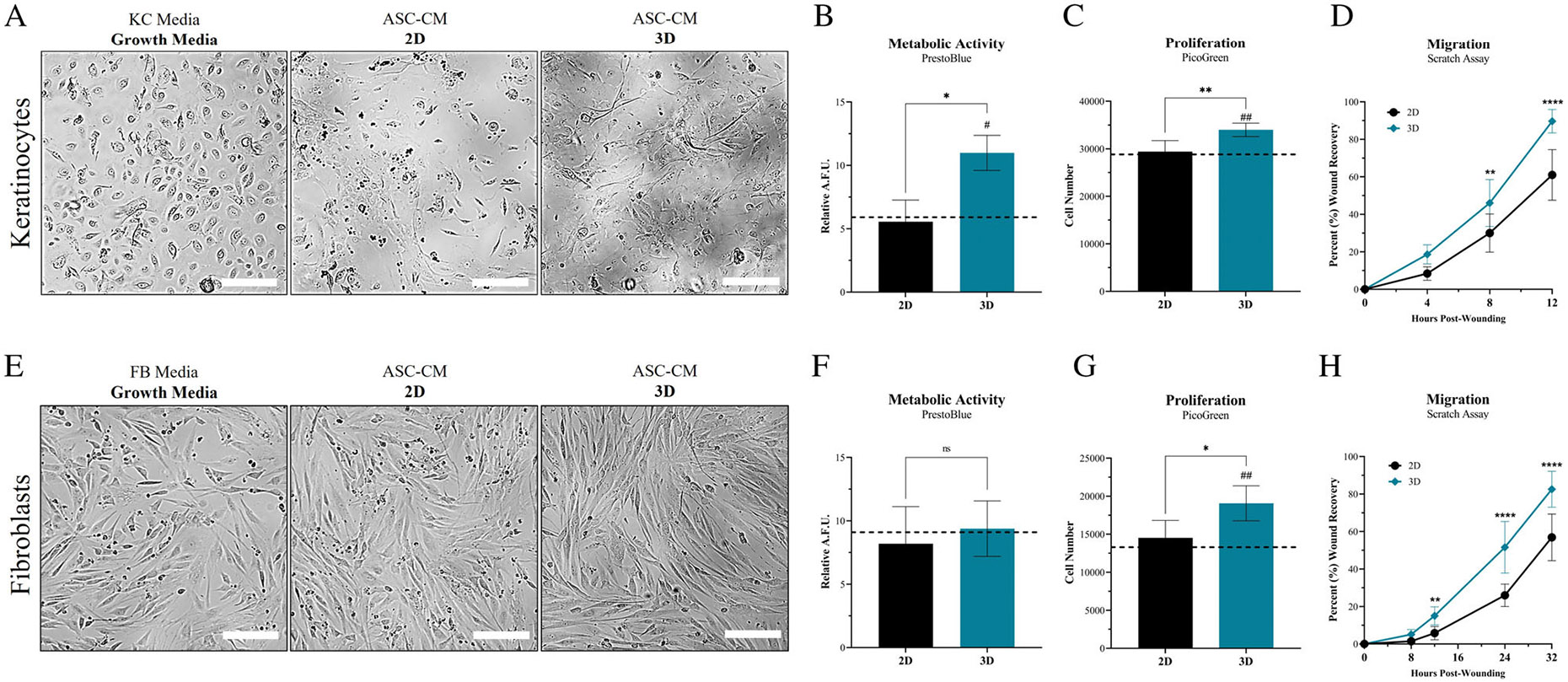
ASC secretome from tissue-mimetic culture enhances KC and FB activity. ASCs seeded at ‘P2’ within the 3D hydrogel system or continuously subcultured for 2 weeks in traditional 2D culture until reaching ‘P5’. The ‘P5’ ASCs were used for characterisation in 2D and ‘P5’ passage-equivalent were used for 3D. ASC-CM was collected from the ‘P5’ and ‘P5’ passage-equivalent ASC cultures, for 2D and 3D, respectively. ASC-CM from 2D and 3D was then used to treat KCs (A–D) and FBs (E–H). (A, E) KCs and FBs were assessed for morphological, (B, F) metabolic, (C, G) proliferative and (D, H) migratory changes. Metabolic activity was quantified via PrestoBlue and then standardised to relative fluorescence of Hoechst 33342 per 96-well, to provide an average R.F.U. value. Proliferative activity was quantified via PicoGreen and then average cell number per 96-well was determined. Average values for KCs and FBs treated with standard growth media is denoted by dashed line (Black). Metabolic and Proliferative activity was performed with five replicates (*n* = 5). Migratory activity was assessed via scratch assay recovery. The voided space created by a pipette tip was evaluated for recovery of area via migration of KCs and FBs. Whole well images were acquired and the recovery area of three different locations per well were averaged. Migration samples were performed in triplicate (*n* = 3) for a total of nine images per treatment group. Average area closed/recovered are denoted for each time point for KCs and FBs after treatment with ASC-CM from 2D (Black Circles) or 3D (Teal Diamonds). Significance is denoted as *p < 0.05, **p < 0.01 and ****p < 0.0001, and ^#^*p* < 0.05 or ^##^*p* < 0.01 for 3D comparison relative to media control. Error bars are standard deviation. One-way ANOVA was used for Metabolic and Proliferative assays. Two-way ANOVA was used for Migratory assay. Scale bar = 200 μm.

To determine the significance of utilising secretome from ASC populations in 3D rather than 2D, the functional capacity of ASC-CM was assessed for its ability to modulate the activity of secondary cell populations involved in wound healing. KCs and FBs were treated with ASC-CM and evaluated for changes in metabolic, proliferative and migratory activity. KCs treated with ASC-CM exhibited spindle-shaped morphological changes relative to culture with KC-GM. KCs treated with 2D ASC-CM had <50% of the KC population undergoing spindle-shaped changes, whereas >50% of KCs treated with 3D ASC-CM demonstrated spindle-shaped morphological changes (Figure 6A). Moreover, KCs treated with 2D ASC-CM had substantially more cell debris, relative to 3D. Stratification and layering of KCs was also observed after treatment with 3D ASC-CM (Figure 6A). Conversely, there were less morphological changes to FBs after treatment with ASC-CM observed. Although, FB-GM treated FBs exhibited smaller cell size and spindle protrusion length overall. The 3D ASC-CM treated FBs demonstrated a more homogenous population of spindle-shaped cells, both in size and morphology, relative to both FB-GM and 2D ASC-CM treated cells. (Figure 6E). KCs treated with ASC-CM from 3D had a significant increase in metabolic and proliferative activity, relative to ASC-CM from 2D (Figure 6B,C). Whereas 3D ASC-CM only significantly increased the proliferative activity of FBs and not metabolic activity, relative to 2D (Figure 6F,G).

The ability for ASC-CM to modulate the migratory capacity of KCs and FBs was then assessed via evaluation with a scratch assay (Figure 6D,H). KCs exhibited the quickest recovery with treatment of ASC-CM from 3D, with regions of complete recovery of the scratched area within 12 h. KCs exhibited ~90% recovery of the scratched area by 12 h post-treatment with ASC-CM from 3D. Whereas KCs exhibited only ~60% recovery of the scratched area at 12 h post-treatment with ASC-CM from 2D (Figure 6D). Similarly, FBs exhibited the quickest rate of recovery when treated with ASC-CM from 3D, though migrated more slowly relative to KCs. FBs had ~83% recovery at 32 h post-treatment with 3D ASC-CM; whereas, FBs had only ~57% recovery at 32 h post-treatment with 2D ASC-CM (Figure 6H). Overall, ASC-CM from 3D demonstrated a significant increase in ability to enhance the rate of recovery via enhanced migratory activity of both KCs and FBs at multiple timepoints throughout the experiment, relative to 2D ASC-CM (Figure 6D,H).

## 4 ∣ DISCUSSION

The multipotent and highly secretive nature of MSCs provide a unique opportunity for advancing the field of regenerative medicine and wound care. However, limitations of current in vitro 2D expansion methodologies currently hinder the growth of MSC-based research and development. In the unnatural and harsh environment of traditional 2D culture systems, MSCs lose their multipotent ‘stem-like’ features, become non-viable and senescent and subsequently decrease their production of regenerative secretory products.^52-54^ Thus, a shift towards 3D culture systems that more closely mimic native in vivo tissue environments are being investigated. Recently, investigations into microcarriers, spheroids/organoids and 3D scaffolds or hydrogel culture systems have been investigated.

There have been a number of studies demonstrating the benefits of 3D culture of MSC-like populations relative to traditional 2D culture and how the culture system effects MSC phenotype. Moreover, the importance of mechanical regulation of MSC phenotype is known. However, ‘3D culture’ and ‘tissue-mimetic’ culture are broadly used terms. Microcarrier systems do allow for enhanced expansion of stem cells, but they are more pseudo-3D and are not tissue-mimetic in nature. The production of native matrix components with cells in 3D spheroidal and organoid culture has demonstrated enhanced abilities for generating MSCs that are more viable and maintain ‘stem-like’ properties, though they do not produce a native tissue-mimetic environment and typically require a large-scale bioreactor system for continuous culturing.^67,68^ Moreover, mass transport and diffusional constraints limit the size and utility of 3D spheroids/organoids for many applications.^67,68^ Recently, tissue engineered 3D hydrogel systems have provided advantages towards producing a tailorable tissue-mimetic system that improve the viability of ‘stem-like’ cells and allow for greater control over differentiation potential of MSCs in culture.^69,70^ However, many 3D hydrogel systems can be limited in size due to diffusional constraints, such as that seen with gels that are poured/moulded/casted which lack a porous architecture, or are derived of a bioactive substrate that ultimately induces differentiation of MSC populations, which results in lack of clarity of the role of the mechanics. To date, there is yet to be any studies investigating how the culture of ASCs within a bioinert but mechanically analogous system effects ASC populations and their secretome in the context of wound healing. Notably, previous studies have yet to utilise a 3D hydrogel system to assess the relationship between senescence and ‘stem-like’ properties of ASC populations to changes in ASC secretive bioactivity.

In this study, we utilise a ‘bioinert’ 3D hydrogel system to improve in vitro culture conditions of ASCs. The porous microarchitecture of this hydrogel system is unique and allows for cellular migration and proliferation within the hydrogel. Notably, cells are seen within the porous channels attaching and creating an interconnected network (Figure 1B). Moreover, the ability to effectively permit the transport of fluids and small molecules within a 3D system is critical to effectively allow nutrient and waste exchange.^70-72^ Thus, the porous nature of this system permits mass transport (Figure 1A) and the easy collection of cellular-derived biologics, such as EVs and proteins, within the secretome (Figures 4 and 5). Therefore, this system was selected because it provided an improved culture environment for ASCs over traditional 2D culture.^73^ Additionally, this 3D system enabled more efficient isolation of cellular byproducts (i.e., biologics) relative to non-porous hydrogel systems, which ultimately lack the capacity for long-term culture and biologics collection that would be necessary for future clinical therapies. For the context of this study, a hydrogel that was mechanically similar to native adipose tissue was generated (~3 kPa; Figure S3).^51,66,73-75^ Additionally, the ‘bioinert’ nature of the hydrogel allowed for the direct observation of the role of the mechanical, dimensional and architectural properties of the 3D system on ASC senescence and phenotype.

Senescent cell populations, specifically stem cells, have been shown to detrimentally alter tissue healing processes by secreting factors known to inhibit angiogenesis, exacerbate inflammation, increase oxidative stress and induce senescence in surrounding cell populations.^58-61^ Moreover, the secretome of senescent ASCs has been shown to directly hinder wound healing activity in secondary cell populations, including the angiogenic response.^61^ Therefore, limiting the induction of senescence in ‘stem-like’ populations is a critical area of research to improve the regenerative and wound healing capabilities of MSC-like cells. Although progression towards senescence in 2D culture systems is well-known,^53^ the significance of ASC senescence on the wound healing capacity of the secretome and whether 3D culture modulates this activity is still under investigation.

In this study, culture of ASCs within a traditional 2D system resulted in a significant increase in senescent cells relative to the initial ‘P2’ ASC populations, whereas 3D culture did not (Figure 2A-C). It is important to note the timeframe of this study of 14 days (or 3 passage events in 2D), as this is a common timeframe found in studies in literature, and is often the minimum required expansion time in vitro to achieve adequate MSC numbers for clinical therapies. The observed differences in senescence for 3D ASCs will need to be further investigated to determine the exact mechanism and dynamics of the ASC senescent populations. Ultimately, this data shows that the prevalence of unfavourable senescent ASC populations can be significantly reduced by culturing ASCs in a tissue-mimetic 3D system, without the need of added supplements or other biomodulatory factors. To our knowledge, this is the first demonstration of a ‘bioinert’ 3D hydrogel culture system that possibly prevents, or significantly delays, the progression towards in vitro senescence of an MSC population without an added supplement.

The ability to maintain a ‘stem-like’ phenotype is also imperative to the overall regenerative capacity of ASC populations, with terminal differentiation into cell populations with different secretomes potentially ‘contaminating’ the secretive composition of the ‘stem-like’ populations. Upon culture of ASCs within the ‘bioinert’ 3D system, ASCs retained their expression of MSC ‘stem-like’ surface and phenotypic markers to a greater extent than 2D, including expression of key growth factors and immunomodulatory compounds (Figure 3A-C). Denoting a possible protective role of this tissue-mimetic 3D system from MSC phenotypic changes, in part, potentially due to the softer mechanical properties and 3D architecture. Future experiments will aim to investigate the difference between this ‘bioinert’ system and an analogous system with additives to generate a more tissue-mimetic system and further augment ASC regenerative capabilities.

Overall, this study reiterates the detrimental effects of traditional 2D culture systems on the ‘stem-like’ properties of ASCs over the course of only three passaging events (14 days). As previously mentioned, many studies in literature use MSC populations typically in the passage 3–6 range for experiments, meaning there is likely variability in the phenotype of these populations, both between studies as well as within the same study. This study provides insight into future design consideration for an MSC culture expansion system and the potential importance of tissue mechanics ad MSC phenotype. Ultimately, biomedical research with MSCs often aims to exploit their ‘stem-like’ properties and characteristics, where loss of ‘stemness’ has been shown to decrease the overall regenerative properties of MSCs.^76,77^ Thus, the ability to improve the retainment of MSCs with higher ‘stem-like’ populations is desirable for a multitude of applications, including cell therapies, regenerative tissue engineering and production of secreted biologics.

To evaluate the significance of culturing ASCs in a tissue-mimetic 3D system rather than traditional 2D on the secretome composition, ASC-CM was collected, analysed and utilised in conditioned media studies with KCs and FBs. Although characterisation of specific ASC secretome components have previously been assessed in different ‘primed’ 2D and 3D systems (e.g., increased secretion of VEGF in fibrin-based gels or increased HGF secretion in 3D spheroids),^78-80^ direct comparison of secretome differences between 2D culture and a ‘bioinert’ 3D hydrogel system have yet to be investigated (i.e., direct investigation into role of 3D mechanics and architecture).

First, ASC-CM was evaluated for the relative composition of key secreted proteins that are important for wound healing and regeneration. Our data demonstrated that ~15% of the proteins tested were significantly increased in 3D (Figure 4), whereas the remaining proteins demonstrated no significant changes. This suggests that the relative composition of soluble proteinaceous factors secreted from ASCs is altered within the 3D hydrogel system. Additionally, this data may suggest a possible increase in total secreted protein production, though more studies are needed to assess global protein production. Notably, solubilised proteins have been shown to directly augment the bioactivity of cellular populations, including the wound healing activity of fibroblasts and keratinocytes.^81-83^ Therefore, this 3D system demonstrates a potential means to produce solubilised proteins for regenerative therapies. Total protein concentration was attempted but due to phenol red within the media, QuickDrop spectrophotometry and BCA analysis were not possible. Future studies will look to optimise utilisation of phenol-free media and assess total proteome.

Similarly, the ASC-CM was further evaluated for antioxidant activity. Increased oxidative activity is one of the many compounding factors that can hinder the tissue regenerative response and promote the progression towards non-healing wounds and tissue.^84,85^ Therefore, antioxidants are critical compounds in the wound healing and tissue regeneration processes, with antioxidant treatments previously demonstrating the capacity to improve the wound healing process.^84-86^ Stem cells are known to secrete antioxidants,^87^ though upon becoming senescent stem cells have demonstrated a shift in the secretion of oxidants relative to antioxidant compounds. In this study, ASC-CM from the 3D system exhibited a significantly higher level of total antioxidant activity relative to 2D (Figure S5). As we have previously stated, senescent stem cells have previously been associated with changes in oxidative activity and have been shown to induce upregulation of oxidative damage in surrounding cell populations, thus there is potentially a relationship between the relative senescence and the relative antioxidative/oxidative balance in 2D and 3D culture systems.^58-60^ Further studies investigating the dynamic relationship between senescence, stemness and antioxidant/oxidant activity are warranted to elucidate any potential connection.

Another form of cargo secreted by ASCs other than antioxidants and solubilised proteins are EVs. In general, EVs consist of a myriad of vesicle-like particles such as apoptotic bodies (500–5000 nm), microvesicles (MVs; 200–1000 nm) and exosomes (Exo; 25–250 nm).^88,89^ EV have garnered a lot of interest of late, due to the diverse array of biomodulatory cargo that they can carry. Depending on the EV type and origin, EVs can proteins, small molecules and nucleic acids for MVs or exosomes; whereas apoptotic bodies tend to secrete chromatin and organelles.^88-90^ Moreover, the relative composition of the contents within EVs have been shown to change in a state-dependent manner.^91^ Thus, the highly adaptive nature of stem cells are known to secrete a variety of compounds within EVs; specifically, the exosomal fraction of ASCs have demonstrated the ability to enhance the rate of wound healing and tissue regeneration.^31,92^

In this study, similar to the total protein fraction, the relative concentration of EVs was significantly increased in the ASC-CM from our 3D system (Figure 5A). Suggesting that ASCs potentially favour secretion of EVs in 3D (and in vivo) and/or are more secretive globally overall in 3D, relative to 2D. Moreover, the size distribution data suggests that the EVs evaluated in this study tended to be smaller and more likely exosomal or MV in nature. The subsequent in vitro tracking assays validated the ability for KCs and FBs to endocytose ASC-derived EVs. Upon internalisation into the cytoplasm of cells, EV/Exosomes have been shown to modulate cellular signalling.^90,92^ Moreover, as observed in this study, a notable change in cellular morphology is demonstrated to a greater extent is cellular populations that endocytosed more EVs (Figure S6), denoting a potential key role of EVs in wound healing physiology that should be further explored. Therefore, one possible mechanism of action of ASC secretome modulation of wound healing activity is via the controlled secretion of EV/Exosomes.^92,93^ Further studies investigating the composition of EV/Exosomes contents between 2D and 3D would provide a better understanding not only in total secretive activity, but the nature of that secretive activity and composition of biomodulatory cargo. To our knowledge, this is the first holistic demonstration of how the secretory production of soluble proteins, antioxidants and EV/exosomes from ASCs can be further enhanced within a tissue mimetic hydrogel system, potentially due to the improved ASC phenotype within the system.

Lastly, to assess the significance of the altered ASC secretome composition on wound healing capacity, ASC-CM co-culture assays were performed to assess for ‘functional changes’ in the ASC-CM (Figure 6). For both KCs and FBs, proliferative activity was significantly increased when treated with ASC-CM from 3D culture, but only KCs exhibited an increase in metabolic activity after treatment with ASC-CM. Similar to previous literature, ASC-CM from traditional 2D culture did expedite the rate recovered scratch area of KCs and FBs, relative to control. However, 3D ASC-CM further enhanced the rate of recovery for KCs and FBs, relative to traditional 2D ASC-CM. Therefore, the augmentation of ASC secretory composition within the 3D hydrogel system resulted in improved functional activity of KCs and FBs, including both proliferation and migration. Unexpectedly, KCs and FBs treated with ASC-CM, specifically ASC-CM from 3D culture, exhibited notable alterations in cellular morphology. The morphological changes seen in secondary cell populations warrants further investigation into what changes are occurring in the KCs and FBs after ASC-CM treatment and what secretome components are driving this activity. However, the increased migratory activity and spindle-shaped morphology (also seen in the in vitro EV tracking assay) suggests a possible differentiation and/or maturation towards a more migratory/mesenchymal phenotype; notably, KCs are known to undergo an epithelial-to-mesenchymal transition (EMT) during re-epithelialization of wounds.^45^

Although ASCs and ASC-CM from 2D culture have previously been shown to modulate wound healing activity of KCs and FBs, this is the first direct comparison of how a tissue-mimetic hydrogel system for ASCs can further enhance the wound healing capacity of the ASC secretome. Further investigations into the signalling dynamics are warranted, however one possible explanation is that retainment of ‘stem-like’ properties in 3D results in greater retainment of more secretive ASC populations overall, while subsequent prevention of senescence results in a secretome composition that is more regenerative. Previous studies have demonstrated the important role of mechanotransduction in ‘stem-like’ cell phenotype and how substrate mechanics can directly affect gene expression via mechanoepigenetics.^49,94,95^ Future studies will aim to investigate the factors driving the benefits demonstrated by 3D culture in this study and aim to determine whether 3D culture modulates the quality and/or quantity of ASC secretory compounds.

Limitations of this study include the utilisation of one ASC donor source. Ideally, future studies will include multiple donor sources to make sure differences seen are not donor-specific. However, since the study directly compared 2D versus 3D culture and not the overall ASC-CM efficacy, the direct comparison between the two systems is reasonable because the relative donor-specific attributes would affect both the 2D and 3D systems equally. Of note, since the goal of this study is to demonstrate the possible utility of an acellular biologic therapy derived of allogeneic ASCs for a wound therapy, a younger and healthier source of ASCs, and an older source of keratinocytes and fibroblasts was desired. Another limitation is the lack of breadth of the proteome array. Although this proteome array provides more information than individual ELISAs, this array only tested for 55 secreted proteins. Therefore, it is not an accurate depiction of the entire proteome. However, in this study we utilise a number of supplementary assays of ASC-CM characterisation to corroborate the differences seen in the proteome array. Future studies will investigate the key proteins in ASC-CM that resulted in augmented KC and FB activity. One possible method will be to perform mass spectroscopy analysis to provide a holistic perspective of the ASC-CM proteome in 2D and 3D. Future studies will also investigate the composition of the EV/Exosomes and provide a clearer perspective of the dynamic changes occurring in ASC secretive activity. Ultimately, 3D in vitro and in vivo wound models will be the subject of future studies after demonstrating the validity of this tissue-mimetic system. However, performing 2D models did allow the authors to directly assess the relative effect of the ASC secretome on individual functions for each cell line separately. Lastly, performing a study to directly compare this system with other 3D culture systems widely used in biomedical research will provide a better understanding of the relative benefits of this system.

As previously discussed, bioactive compounds coordinate and bridge a variety of tissue reparative processes and hold immense regenerative capabilities. Studies have shown that ASCs are capable of modulating secondary cell populations via the secretion of bioactive compounds such as growth factors, cytokines, exosomes, and microvesicles in an autocrine, paracrine, and endocrine manner. Thus, the dynamic and diverse milieu of biomodulatory compounds secreted from ASCs offers a unique approach for tailored clinical therapeutics.^47^ However, there is still much about the secretive bioactivity of ASC populations that is unknown. The role of culture expansion, viability, senescence, ‘stem-like’ phenotype, and other stochastic processes on the overall composition and functionality of the ASC secretome remains largely unexplored. With the growing interest for regenerative acellular biologics due to limitations of autologous and allogeneic cell-based therapies, this study aimed to introduce how a tissue-mimetic 3D hydrogel system improves the culture expansion of ASCs and the subsequent production of acellular biologics that can augment wound healing activity of secondary cell populations.

## 5 ∣ CONCLUSION

In conclusion, this study was a ‘proof-of-concept’ study meant to serve as a template for future study designs and illustrate the benefits of culturing MSCs within a porous tissue-mimetic 3D system that mechanically relates to their native tissue (rather than 3D spheroids or microcarriers). The results of this study shed further insight into the heterogeneity of MSC populations and the effect of inadequate culture conditions on the regenerative capacity of the cells. Moreover, this study will lay the foundation for future studies looking to tailor the properties of this 3D system to generate a more tissue-mimetic system via incorporating biomodulatory compounds to enhance the control over phenotype and secretive bioactivity. For example, after establishing the beneficial effects of this ‘bioinert’ 3D hydrogel system on ASC populations, extracellular matrix (ECM) substrates (e.g., fibrin, collagen, gelatin, elastin), biologics (e.g., growth factors or cytokines) and environmental stimuli (e.g., hypoxia) can be introduced as stimuli into the 3D system to further modulate ASC activity. Thus, this system provides means to generate customizable ASC-derived biologics for future regenerative therapeutic modalities for a variety of applications, including integration into current wound healing modalities.

## Supplementary Material

Supp Info

Supplementary Table 1

Supplementary Table 2

Additional supporting information can be found online in the Supporting Information section at the end of this article.

## Data Availability

The authors collectively declare that all data supporting the findings of the presented work are available within the paper and its supplementary information files.

## Abbreviations:

ASC: adipose-derived mesenchymal stem/stromal cells
ASC-CM: ASC conditioned media
CM: conditioned media
EMT: epithelial-to-mesenchymal transition
EV: extracellular vesicles
FB: fibroblast
FB-GM: fibroblast growth medium
KC: keratinocytes
KC-GM: keratinocyte growth medium
MSC: mesenchymal stem/stromal cells
MSC-GM: mesenchymal stem cell growth medium
MV: microvesicles
NTA: nanoparticle tracking analysis
PEG: polyethylene glyco
R.F.U.: relative fluorescent unit
UA: uric acid
