# Supplementary material for: Tissue-mimetic culture enhances mesenchymal stem cell secretome capacity to improve regenerative activity of keratinocytes and fibroblasts in vitro
Source: Wound Repair Regen. Author manuscript; Available in PMC 2025 Jul 31. (PMC12312442; doi:10.1111/wrr.13076)
Supplement: Supp Info [file NIHMS2097587-supplement-Supp_Info.pdf]

Supplemental Methods and Data

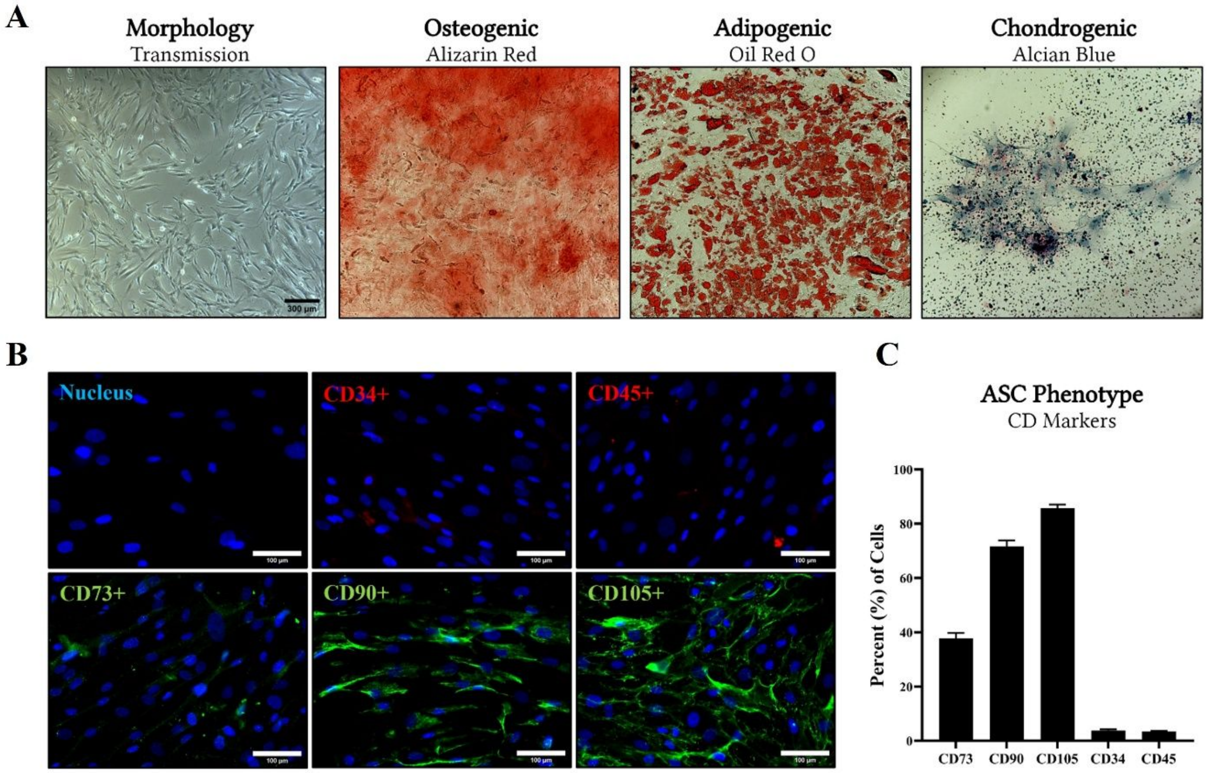

Supplemental Figure 1: Characterization of ASC Multipotency and Phenotype. Adipogenic, chondrogenic, and osteogenic trilineage differentiation potential of ASCs was performed via culture with differentiating media, according to the manufacturer's instructions. Adipogenic differentiation was performed using hMSC Adipogenic Differentiation BulletKit™ (Lonza; Cat. #PT-3004). Chondrogenic differentiation was performed using hMSC Chondrogenic Differentiation Medium BulletKit™ (Lonza; Cat. #PT-3003), and was supplemented with TGF-β3 (Lonza; PT-4124) at a concentration of 10ng/mL. Osteogenic differentiation was performed using hMSC Osteogenic Differentiation Medium BulletKit™ (Lonza Cat. #PT-3002). After conclusion of adipogenic differentiation, cells were fixed in 4% paraformaldehyde and stained with Oil Red O (ScienCell; Cat. #0843), per the manufacturer's protocol. For chondrogenic differentiation, cells were maintained in MSC-GM until ~100% confluency was reached. Media was then switched to chondrogenic differentiation media for ~21 days, with media changes every 2-3 days. After conclusion of chondrogenic differentiation, cells were fixed in 4% paraformaldehyde and

stained with Alician Blue (ScienCell; Cat. # 8378), per the manufacturer's protocol. For osteogenic differentiation, cells were maintained in MSC-GM until ~70% confluency was reached. Media was then switched to osteogenic differentiation media for ~21 days, with media changes every 3-4 days. After conclusion of osteogenic differentiation, cells were fixed in 4% paraformaldehyde and stained with Alizarin Red S (ScienCell; Cat. # 0223), per the manufacturer's protocol. (A) Mesenchymal "stem-like" characteristics of initial "P1" ASC population was assessed with positive adherence to culture flask and demonstration of spindle-like morphology. Exhibiting trilineage differentiation potential via media challenge with positive staining for Osteogenic, Adipogenic, and Chondrogenic markers. Objective = 10x Scale bar = 300µm. (B) Expression of "stemness" cell surface markers for "P1" ASCs was assessed via immunolabeling and positive staining for CD73, CD90, and CD105, and less than 5% positive for CD34 and CD45. Samples were performed in quadruplicate (n=4) for quantification. Counterstained with Hoechst. Objective = 20x. Scale bar = 100µm. (C) Quantification of immunolabeled "P1" ASCs.

For Peer Review

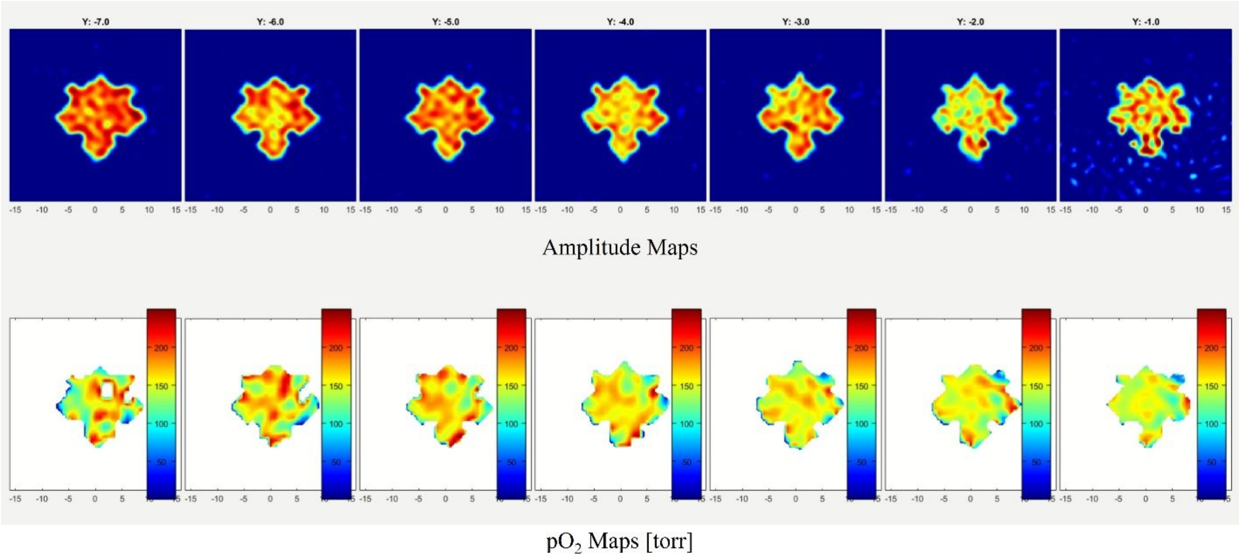

Supplemental Figure 2: Oxygen Distribution Throughout 3D Hydrogel. Courtesy of O2M Technologies, the ability of nutrients and gases to distribute throughout the 3D system was indirectly assessed via a conjugated oxygen isotope and magnetic resonance, which demonstrated relatively homogenous distribution throughout the hydrogel. Ambient oxygen should have a partial pressure of ~160 Torr, which would be in the yellow-orange color range in the dataset provided. As we can see, the oxygen distribution and partial pressure hits these levels of oxygen in less than 1 hour. This data denotes how gas can quickly equilibrate within the 3D hydrogel system in this study.

For Peer Review

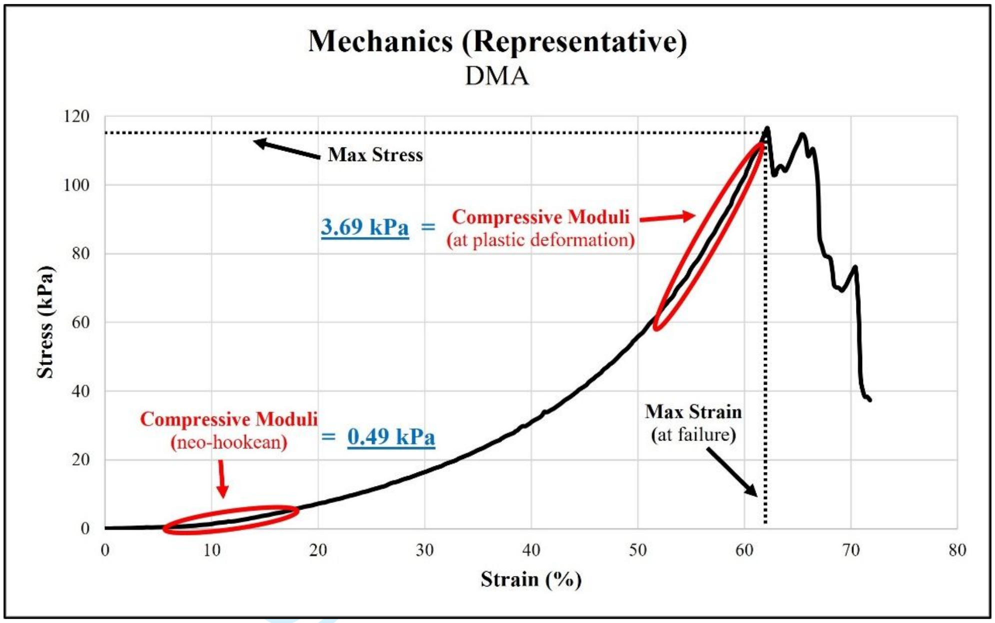

Supplemental Figure 3: Mechanical and Physical Characterization of Hydrogel System. 3D hydrogels were bioprinted that were 1.2-mm (thickness) but still contained the macro- and micro- architectural design of the full-sized hydrogels that are traditionally 1-cm (length) x 1-cm (width) x 1-cm (thickness). Hydrogels were sterilized, with 10% pen/strep and UV irradiated for 3-hours, followed by submersion in HBSS for 24 hours. Hydrogels were removed and analyzed with a Dynamic Mechanical Analyzer (DMA; TA Instruments, RSA3) setup. A 5-mm biopsy punch was used to isolate a circular hydrogel sample to prevent force-concentrating points within the gel. DMA was performed via a dynamic cylindrical compression analysis with a rate of compression of 0.005-mm/sec. DMA was performed in quadruplicate (n=4) with technical replicate (x2) for each gel, for a total of 8 samples processed. Max stress was taken at the point of failure (plastic deformation) and max strain was considered the strain percent at the point of failure (plastic deformation). Two different compressive elastic moduli are reported in literature for hydrogels. However, typically most compressive moduli for tissue include the modulus at plastic deformation.

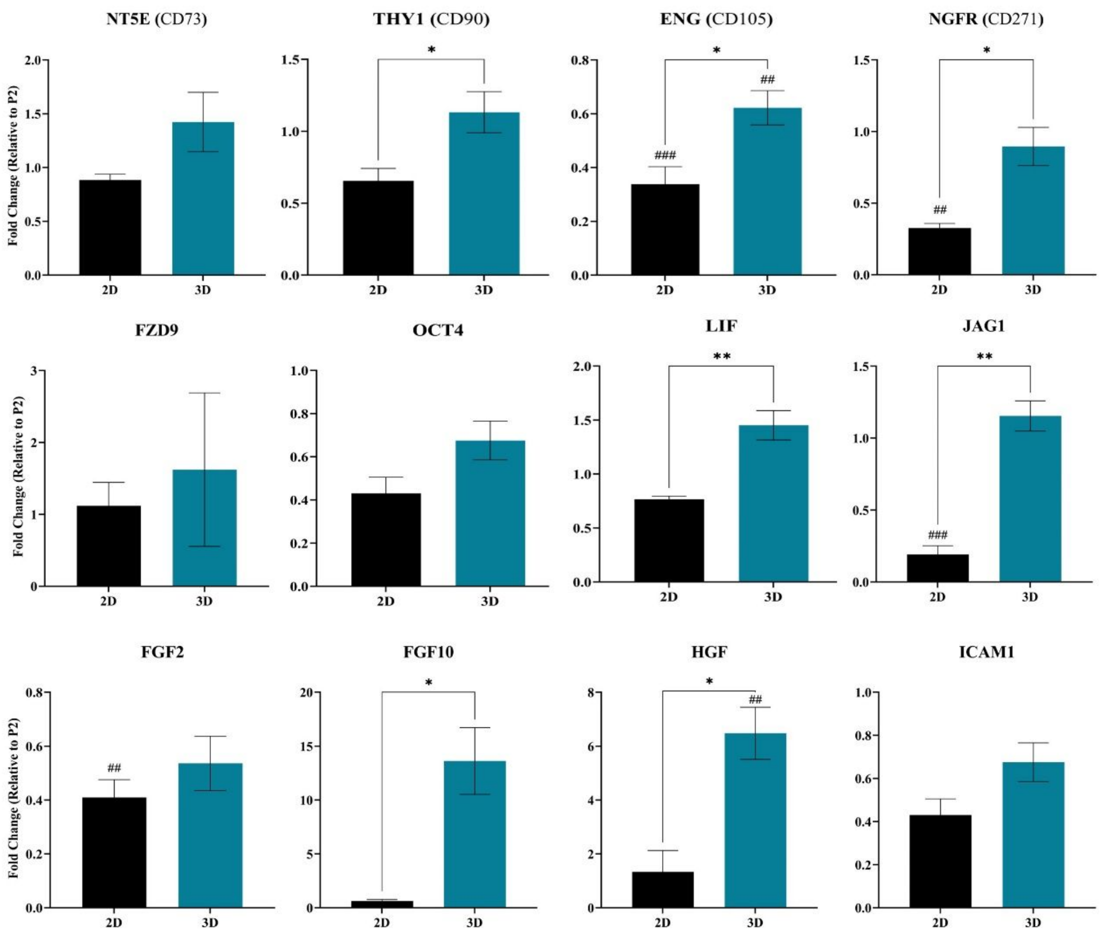

Supplemental Figure 4: Gene Expression Array of MSC "Stem-Like" Phenotype in 2D versus 3D. ASCs isolated from both 2D and 3D culture at "P5" were assessed for expression of key genes via a MSC Phenotyping array. The relative fold change of twelve (12) key genes are displayed for the gene expression of "P5" ASCs in 2D (*Black*) or 3D (*Blue*). Fold change is relative to baseline control "P2" ASCs. Values are normalized to a group of endogenous control genes, that included GAPDH, ACTB, and B2M. RNA analysis was performed in triplicate (n=3). One-Way ANOVA with Tukey's post-hoc was used for statistical analysis. Significance of "P5" 2D or 3D ASCs relative to control "P2" ASCs is denoted with a '#' above the bar and indicates a $p < 0.05$. Differences between 2D and 3D that are significant are denoted as $^*p < 0.05$, $^{**}p < 0.01$, or $^{***}p < 0.001$.

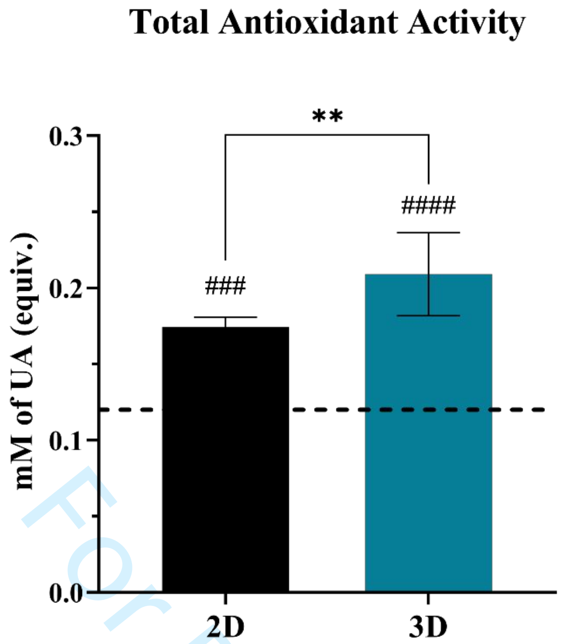

Supplemental Figure 5: Quantification of Relative Antioxidant Activity within ASC-CM. ASC-CM was collected as previously discussed, and antioxidant activity was assessed with a Total Antioxidant Capacity (TAC) Assay kit (Cell Biolabs; Cat. #STA-360). The kit was carried out per the manufacturers protocol. In short, the reduction of copper (II) to copper (I) by antioxidant activity is assessed, with the naturally occurring antioxidant uric acid, used as a control standard for the kit. Antioxidant activity of ASC-CM was therefore measured in mM equivalents of uric acid. Control MSC media was used to determine baseline antioxidant activity of the media without exposure to cells. Assay was performed with technical replicates and biological triplicates (n=3). In short, ASCs seeded at "P2" within the 3D hydrogel system or continuously subcultured for 2 weeks in traditional 2D culture until reaching "P5". The "P5" ASCs were used for characterization in 2D and "P5" passage-equivalent were used for 3D. ASC-CM was collected from the "P5" and "P5" passage-equivalent ASC cultures, for 2D and 3D, respectively. Total antioxidant activity of ASC-CM was measured in uric acid (UA) equivalents and quantified according to kit instructions via plate reader analysis. Assay was performed in triplicate (n=3) and averaged. Error bars are standard deviation. One-way ANOVA with Tukey's post-hoc used for statistical analysis. Significance denoted as Significance is denoted as *p < 0.05, **p < 0.001, ***p < 0.001, and ****p < 0.0001for 2D versus 3D comparison, and $^{###}p < 0.001$ or $^{####}p < 0.0001$ for 2D or 3D comparison relative to control media.

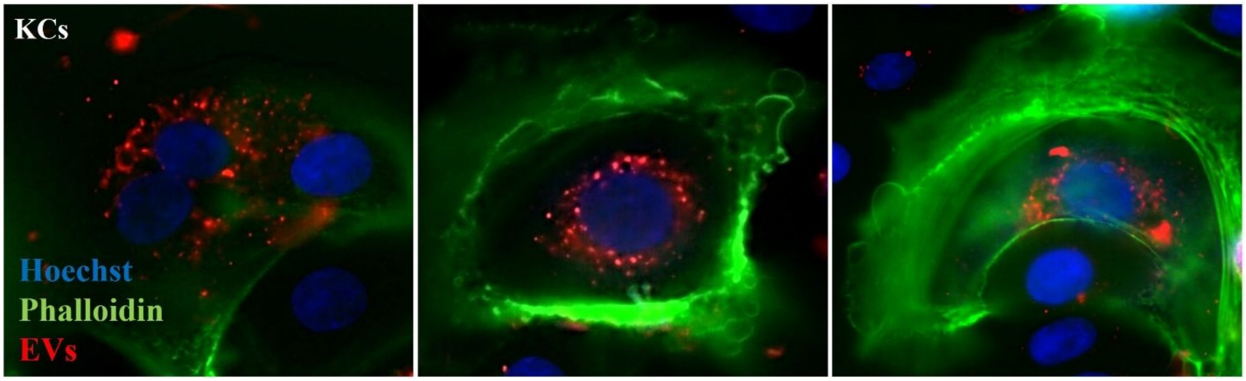

Supplemental Figure 6: Keratinocyte uptake of ASC-derived EVs. EVs that were isolated from ASC conditioned media were labeled with a lipophilic dye, DiI, and then dosed into KC media overnight (~18 hours). KCs were then fixed and stained. Colors include Hoechst 33342 (*Blue*), Phalloidin-AF488 (*Green*), and DiI-labeled EVs (*Red*). KCs taking up larger quantities of DiI-labeled EVs from ASCs can be clearly seen undergoing more substantial cytoskeletal changes (via phalloidin stain) when compared to cells that do not appear to have as many EVs within them. Additionally, EVs can be seen localized in a perinuclear fashion.

For Peer Review
