# Supplementary material for: Tissue-mimetic culture enhances mesenchymal stem cell secretome capacity to improve regenerative activity of keratinocytes and fibroblasts in vitro
Source: Wound Repair Regen. Author manuscript; Available in PMC 2025 Jul 31. (PMC12312442; doi:10.1111/wrr.13076)
Supplement: Supplementary Table 1 [file NIHMS2097587-supplement-Supplementary_Table_1.docx]

| **Gene** | ***2D Fold Change*** | ***3D Fold Change*** | **Gene** | ***2D Fold Change*** | ***3D Fold Change*** | **Gene** | ***2D Fold Change*** | ***3D Fold Change*** |
| --- | --- | --- | --- | --- | --- | --- | --- | --- |
| *ABCB1* | n/a | n/a | *GDF7* | n/a | n/a | *NUDT6* | 1.523 | 1.924 |
| *ACTA2* | 0.902 | 0.857 | *GTF3A* | 0.967 | 1.147 | *PDGFRB* | 1.445 | 1.950 |
| *ALCAM* | 0.680 | 0.600 | *HAT1* | 0.994 | 1.194 | *PIGS* | 0.765 | 1.127 |
| *ANPEP* | 0.733 | 0.576 | *HDAC1* | 0.858 | 0.879 | *POU5F1 (OCT4)* | 0.431 | 0.675 |
| *ANXA5* | 0.937 | 0.830 | ****HGF*** | ***1.335*** | ***6.481*** | *PPARG* | 1.199 | 1.568 |
| *BDNF* | 0.593 | 0.460 | *HNF1A* | n/a | n/a | *PROM1* | n/a | n/a |
| *BGLAP* | 1.640 | 1.105 | *ICAM1* | 0.354 | 1.970 | *PTK2 (FAK)* | 0.743 | 0.929 |
| *BMP2* | 0.735 | 0.829 | *IFNG* | n/a | n/a | *PTPRC* | n/a | 1.042 |
| ****BMP4*** | ***0.396*** | ***2.753*** | *IGF1* | 0.339 | 1.097 | *RHOA* | 1.120 | 0.865 |
| *BMP6* | 0.142 | 0.371 | *IL10* | n/a | n/a | *RUNX2* | 1.399 | 1.645 |
| *BMP7* | n/a | n/a | *IL1B* | 0.133 | 0.145 | *SLC17A5* | 1.011 | 1.702 |
| *CASP3* | 1.035 | 1.109 | *IL6* | 0.437 | 1.180 | *SMAD4* | 0.969 | 1.046 |
| ****CD44*** | ***1.042*** | ***0.764*** | *INS* | 2.938 | 0.561 | *SMURF1* | 0.699 | 0.898 |
| *COL1A1* | 0.818 | 1.154 | *ITGA6* | 0.330 | 0.560 | *SMURF2* | 0.851 | 0.895 |
| ****CSF2 (GM-CSF)*** | ***5.298*** | ***12.761*** | *ITGAV* | 0.805 | 0.916 | *SOX2* | n/a | n/a |
| *CSF3 (G-CSF)* | 0.789 | 0.782 | *ITGAX* | n/a | n/a | ****SOX9*** | ***1.180*** | ***2.746*** |
| *CTNNB1* | 1.421 | 1.133 | *ITGB1* | 0.929 | 0.725 | *TBX5* | 2.239 | 1.768 |
| *EGF* | 3.681 | 3.635 | ****JAG1*** | ***0.192*** | ***1.154*** | *TERT* | n/a | n/a |
| ****ENG (CD105)*** | ***0.338*** | ***0.623*** | *KAT2B* | 0.974 | 1.333 | *TGFB1* | 0.820 | 0.939 |
| *ERBB2* | 1.337 | 1.437 | *KDR (VEGFR3)* | 0.859 | 1.284 | *TGFB3* | 0.734 | 1.936 |
| ****FGF10*** | ***0.632*** | ***13.620*** | *KITLG* | 1.139 | 1.166 | ****THY1 (CD90)*** | ***0.655*** | ***1.133*** |
| *FGF2* | 0.409 | 0.536 | ****LIF*** | ***0.765*** | ***1.452*** | *TNF* | 0.757 | 0.841 |
| *FUT1* | 0.263 | 0.622 | *MCAM* | 0.782 | 0.777 | ****VCAM1*** | ***3.331*** | ***6.837*** |
| *FUT4* | 1.182 | 1.250 | *MMP2* | 1.527 | 2.238 | *VEGFA* | 0.838 | 0.638 |
| *FZD9* | 1.118 | 1.623 | *NES* | 2.516 | 2.276 | ****VIM*** | ***2.375*** | ***0.927*** |
| *GDF15* | 1.446 | 1.871 | ****NGFR (CD271)*** | ***0.326*** | ***0.895*** | ****VWF*** | ***10.286*** | ***36.942*** |
| ****GDF5*** | ***6.847*** | ***2.074*** | *NOTCH1* | 0.887 | 1.029 | *WNT3A* | n/a | n/a |
| *GDF6* | 1.036 | 1.326 | *NT5E (CD73)* | 0.884 | 1.423 | ****ZFP42*** | ***1.735*** | ***0.161*** |

**Supplementary Table 1: Human MSC Phenotyping Array Targets**

****Denotes Significant Difference Between 2D and 3D with a p < 0.05***
