# Supplementary material for: Tissue-mimetic culture enhances mesenchymal stem cell secretome capacity to improve regenerative activity of keratinocytes and fibroblasts in vitro
Source: Wound Repair Regen. Author manuscript; Available in PMC 2025 Jul 31. (PMC12312442; doi:10.1111/wrr.13076)
Supplement: Supplementary Table 2 [file NIHMS2097587-supplement-Supplementary_Table_2.docx]

| **Human Proteome Profiler Array** | | |
| --- | --- | --- |
| Activin A | FGF-7/KGF | PD-ECGF |
| ADAMTS-1 | GDNF | PDGF-AA |
| Angiogenin | GM-CSF | PDGF-AB/PDGF-BB |
| Angiopoietin-1 | HB-EGF | Persephin |
| Angiopoietin-2 | HGF | CXCL4/PF4 |
| Angiostatin/Plasminogen | IGFBP-1 | PlGF |
| Amphiregulin | IGFBP-2 | Prolactin |
| Artemin | IGFBP-3 | Serpin B5/Maspin |
| Tissue Factor/Factor III | IL-1 beta | Serpin E1/PAI-1 |
| CXCL16 | CXCL8/IL-8 | Serpin F1/PEDF |
| DPPIV/CD26 | LAP (TGF-beta 1) | TIMP-1 |
| EGF | Leptin | TIMP-4 |
| EG-VEGF | CCL2/MCP-1 | Thrombospondin-1 |
| Endoglin/CD105 | CCL3/MIP-1 alpha | Thrombospondin-2 |
| Endostatin/Collagen XVIII | MMP-8 | uPA |
| Endothelin-1 | MMP-9 | Vasohibin |
| FGF acidic | NRG1-beta 1 | VEGF |
| FGF basic | Pentraxin 3 | VEGF-C |
| FGF-4 |  |  |

**Supplementary Table 2: Human Proteome Array Targets**
